# Low-Temperature Sintering Inks for Printed Bioelectronics: Materials, Mechanisms, and Emerging Ideas

**DOI:** 10.3390/bios16040206

**Published:** 2026-04-03

**Authors:** Abhijit Bera, Fei Liu, Matthew R. Marander, Ricardo Ortega, Ahmad Mustafa Ahmad Malkawi, Utsav Kumar Dey, Ritinder Sandhu, Tyler P. Collins, Shan Jiang

**Affiliations:** 1 Department of Materials Science and Engineering, Iowa State University of Science and Technology, 528 Bissell Rd, Ames, IA 50012, USA; 2 Department of Aerospace Engineering, Iowa State University of Science and Technology, 528 Bissell Rd, Ames, IA 50012, USA

**Keywords:** printed electronics, low-temperature sintering, conductive inks, biosensors, flexible electronics, metal–organic decomposition inks, nanoparticle inks, bio-integrated electronics

## Abstract

Printed electronics have emerged as a versatile manufacturing platform for next-generation biosensors, enabling on-demand and low-cost fabrication of functional devices on flexible, stretchable, and unconventional substrates. One major challenge in this field lies in the sintering of printed features, as conventional high-temperature processing is incompatible with polymeric substrates and thermally sensitive biological components. Low-temperature sintering inks, typically processed below 200 °C or even at room temperature, have become a critical enabling technology for bio-integrated electronics. This review provides an overview of the current state-of-the-art and key challenges associated with low-temperature sintering inks for printed bioelectronics. We discuss inks based on metal nanoparticles, metal–organic decomposition precursors, metal oxides, chalcogenides, and hybrid material systems. The emphasis is on how ink chemistry, ligand selection, and precursor structure govern rheology, stability, and sintering behavior. In addition, key low-temperature sintering and curing strategies, including thermal, photonic, laser, plasma, microwave, and chemical sintering, are compared in terms of energy delivery, densification mechanisms, and substrate compatibility. Finally, we outline emerging directions towards low temperature and room-temperature sintering inks, and sustainable biobased ink formulations, and discuss their applications for wearable, implantable, and soft biosensing platforms.

## 1. Introduction

Printed electronics encompass electronic devices fabricated using various printing techniques. They have quickly emerged as a transformative manufacturing paradigm for next-generation electronic systems. By enabling on-demand, low-cost, and additive fabrication on flexible and unconventional substrates, printed electronics offer significant advantages over conventional manufacturing approaches [1,2,3]. Traditional electronic manufacturing processes rely heavily on complex, multistep lithographic processes on rigid semiconducting wafers [4]. In contrast, the new printing technology, such as inkjet [5,6], direct ink writing [7], aerosol printing [8], and electrohydrodynamic (EHD) printing [9,10], allows electronic circuits to be patterned directly onto plastics, paper, elastomers, and textiles in a cost-effective and materials-efficient manner [11]. In particular, these capabilities have become important with the rapid growth of wearable and implantable biosensors, epidermal electronics, and soft bio-interfaces, which place stringent demands on materials selection and processing routes that are compatible with temperature-sensitive substrates and bio-functional components [12,13,14,15,16].

In printed electronics, functional materials are typically delivered in the form of liquid ink, consisting of active materials dissolved or dispersed in suitable solvents and deposited onto substrates with high spatial resolution and minimal material waste. As a result, ink formulation plays a central role in determining print quality and device performance. Key fluid dynamic and rheological parameters, including viscosity and surface tension, directly influence droplet formation, ink spreading, and pattern fidelity during printing [16,17,18]. Following deposition, the interaction between the ink and the substrate, as well as morphological evolution during solvent evaporation and post-processing, critically affects the mechanical and functional properties of the printed features. Challenges such as coffee-ring effects, poor film uniformity, and incomplete particle coalescence must be carefully managed through solvent selection, formulation design, and post-processing strategies [19].

Among different printed electronic devices, bioelectronic devices are gaining interest in medical and healthcare systems. Bioelectronic or biosensor device platforms differ from conventional printed electronic circuits because they integrate electronic materials with biological recognition elements and soft bio-interfaces. A typical printed biosensor consists of multiple functional layers, including conductive electrodes (working, reference, and counter electrodes), dielectric or insulating layers, selective membranes or catalytic coatings, and bio-functional layers such as enzymes, antibodies, or ion-selective materials. These layers are commonly fabricated on flexible substrates, including polyethylene terephthalate (PET), polyimide, elastomers, hydrogels, textiles, or biodegradable polymers that can conform to biological tissues [12,20,21]. These elements must operate synergistically to convert biochemical interactions into measurable electrical signals through electrochemical, potentiometric, capacitive, or chemi-resistive transduction mechanisms [22].

Beyond the presence of individual functional layers, the overall device architecture plays a critical role in determining biosensor performance. In printed biosensors, electrode design, including working, reference, and counter electrodes, governs signal stability, noise level, and electrochemical reproducibility [21,23,24,25,26]. The working electrode surface, often modified with nanostructured materials or catalytic coatings, directly influences electron transfer kinetics and sensitivity [27,28,29,30,31,32]. In parallel, the recognition layer (e.g., enzymes, antibodies, ion-selective membranes) provides chemical specificity and dictates selectivity toward target analytes [32,33,34]. The integration of these bio-functional layers with printed electrodes requires careful control of surface chemistry and roughness, which are strongly influenced by ink formulation and sintering conditions [35,36,37,38,39,40].

An encapsulation layer in a biosensor is a protective coating that shields the device from environmental factors (moisture, oxygen, and biofluids) while allowing the target analyte to diffuse to the sensing interface. It plays a crucial role in improving device stability, selectivity, and biocompatibility. As an example, in wearable glucose sensors, a thin PDMS encapsulation layer is commonly used to protect printed electrodes from sweat and mechanical damage while still permitting glucose diffusion to the enzyme-functionalized electrode [41]. Encapsulation layers further define device reliability by protecting the sensor from environmental degradation while maintaining analyte accessibility [42,43,44,45]. For wearable and implantable biosensors, encapsulation must balance permeability, biocompatibility, and mechanical compliance [46,47]. Consequently, printed biosensor architectures represent a tightly coupled system in which electrode materials, bio-recognition elements, and encapsulation layers must be co-designed with low-temperature processing constraints to achieve stable and high-performance sensing.

Unlike conventional microelectronics, biosensor platforms impose strict thermal constraints during fabrication. Many flexible substrates used in wearable or implantable devices exhibit low glass-transition temperatures (often below 150 °C), which limits the allowable thermal budget during processing [48,49,50]. Furthermore, biological recognition elements such as enzymes, antibodies, or nucleic acids can denature or lose functionality when exposed to elevated temperatures or harsh chemical environments [27,51,52]. As a result, fabrication strategies for printed biosensors must maintain the structural integrity of both the substrate and bio-functional components while still achieving electrically conductive electrode networks.

Despite significant advances in printing technologies, the sintering step remains a major bottleneck. While printing itself can be carried out under ambient conditions with high pattern fidelity, achieving low electrical resistivity, strong adhesion, and long-term reliability still depends on effective post-print processing. Conventional sintering processes used in electronic manufacturing often require temperatures exceeding 300–800 °C to remove stabilizing ligands and densify metallic films [53,54]. Such conditions are incompatible with polymer substrates and bio-functional interfaces and may lead to substrate deformation, delamination, or degradation of biomolecules. Consequently, the development of low-temperature sintering inks and alternative curing strategies has become a key enabling technology for printed bioelectronics. These approaches allow conductive networks to be formed at temperatures typically below 200 °C and, in some cases, at room temperature, while maintaining compatibility with soft substrates and biological interfaces [1,2,11]. Biosensing platforms commonly employ flexible substrates such as polyethylene terephthalate, polyimide, polyurethane, elastomers, hydrogels, paper, and textiles. These materials often exhibit low glass-transition temperatures and are incompatible with prolonged thermal exposure. Moreover, biosensors frequently integrate enzymes, antibodies, ion-selective membranes, and hydrogel layers that are thermally labile and chemically sensitive. Low-temperature sintering thus plays a dual role by preserving substrate integrity while maintaining the biological activity essential for sensor performance [15,16,55].

Importantly, “low temperature” does not necessarily imply uniform heating of the entire substrate. Many modern approaches exploit selective and transient heating, in which the printed material absorbs energy more efficiently than the underlying substrate, enabling rapid densification while maintaining a low effective substrate temperature [5,38,54,56,57]. This distinction is especially critical for biosensors and soft electronics, where conductive layers must be integrated alongside insulating dielectrics, stretchable encapsulants, and bio-functional coatings. In many cases, the thermal budget required for sintering dictates substrate selection, limits multilayer integration, and constrains compatibility with roll-to-roll and high-throughput manufacturing [2,5]. In addition, high-temperature sintering can induce delamination, cracking, or reflow of previously deposited layers, complicating the fabrication of complex sensor architectures. Consequently, low-temperature sintering inks, often combined with photonic, laser, plasma, microwave, or chemical sintering techniques, have become central to overcoming these manufacturing constraints [5,14,38,57].

Low-temperature sintering inks are typically formulated using nanoparticles, reactive precursors, or hybrid material systems designed to lower the thermodynamic and kinetic barriers to densification. For printing conductive patterns, silver (Ag) nanoparticle inks remain the most widely used owing to their high intrinsic conductivity and resistance to oxidation, while Cu-based inks are increasingly explored for cost-effective, large-area applications despite challenges related to oxidation and surface passivation [54,58,59,60]. In parallel, particle-free metal–organic decomposition (MOD) inks provide an alternative strategy in which soluble metal complexes decompose directly into metallic films at reduced temperatures [53,61,62].

The performance requirements of printed devices vary widely across applications; however, biosensors impose particularly stringent and multidimensional figures of merit. In addition to low sheet resistance and high electrical conductivity, printed electrodes for biosensing must exhibit electrochemical stability in aqueous and biofluid environments, minimal signal drift, low noise, and compatibility with surface functionalization chemistries. Mechanical durability under bending, stretching, and repeated deformation is essential for wearable systems, while implantable or transient sensors demand biocompatibility and, in some cases, controlled biodegradation [12,13,55]. Beyond biosensors, low-temperature sintering inks enable a broad range of applications, including flexible displays, soft robotics, electronic skins, and human–machine interfaces, where conductivity at low processing temperatures, adhesion to polymeric substrates, resistance to cyclic fatigue, and scalability to large-area manufacturing are critical metrics. The convergence of these requirements has driven extensive research into ink chemistry, sintering mechanisms, and processing strategies capable of delivering high-performance features without exceeding the thermal and chemical limits imposed by soft and bio-integrated electronics.

In addition to achieving high electrical conductivity, electrodes used in biosensors must satisfy several additional requirements that are not typically encountered in conventional printed electronics. These include electrochemical stability in aqueous environments, minimal signal drift during long-term operation, compatibility with surface functionalization chemistries, and mechanical durability under repeated bending or stretching [21,55]. The microstructure of the electrode surface generated during sintering, including particle size, porosity, and grain connectivity, can significantly influence the electrochemical performance and sensitivity of the biosensor [28,35]. Consequently, optimizing sintering conditions is essential not only for achieving high conductivity but also for controlling the electrode morphology that governs analyte transport and signal generation.

From a biosensing perspective, the relationship between ink formulation, sintering conditions, and device architecture ultimately determines sensing performance. From a transduction perspective, printed biosensors rely on diverse sensing mechanisms, including electrochemical (amperometric and potentiometric), capacitive, piezoresistive, and chemi-resistive responses [63,64]. The effectiveness of these mechanisms is strongly governed by the microstructure and surface chemistry of the printed electrodes, which are in turn dictated by sintering conditions [65,66,67]. For example, electrochemical biosensors depend on efficient electron transfer at the electrode–electrolyte interface, where sintering-induced grain connectivity and surface cleanliness directly influence charge transfer resistance [68]. Similarly, capacitive and impedance-based sensors are sensitive to dielectric layer integrity and interfacial defects arising from incomplete precursor conversion or residual organics [69,70].

Importantly, sintering temperature, atmosphere, and energy delivery mode determine not only electrical conductivity but also surface roughness, porosity, and active surface area [71,72]. Highly porous electrodes generated under mild sintering conditions can enhance analyte diffusion and increase electrochemically active surface area, improving sensitivity [73,74]. However, excessive porosity or incomplete ligand removal may introduce noise and signal drift [75]. Therefore, the selection of sintering strategies must be carefully tailored to the targeted sensing mechanism. Parameters such as electrode conductivity, surface roughness, and interfacial chemistry directly influence electron transfer kinetics, catalytic activity, and detection sensitivity. Therefore, understanding how low-temperature sintering strategies affect both the electronic and biochemical components of printed biosensors is essential for designing reliable sensing platforms.

**Figure 1:**
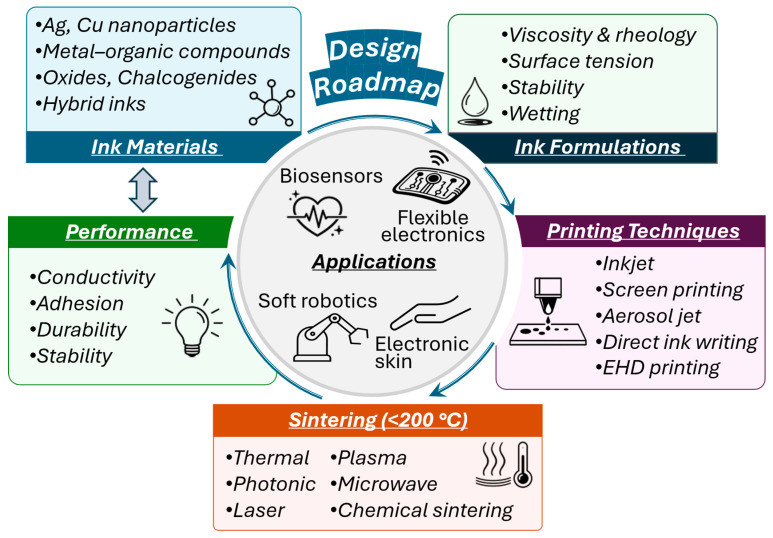
Conceptual diagram showing how ink materials, formulation, printing techniques, and low-temperature sintering govern device performance and enable applications in printed electronics.

In this review, we survey the state of art of low-temperature sintering inks based on metal nanoparticles, metal–organic decomposition precursors, metal oxides, chalcogenides, and emerging material systems (Figure 1). We examine how ink chemistry, ligand selection, and precursor structure influence rheology, stability, sintering behavior, and resulting structure–property relationships. Key low-temperature sintering pathways, including thermal, photonic, laser-assisted, plasma, microwave, and chemical processes, are discussed with emphasis on localized energy delivery and interparticle interactions. Finally, application examples of biosensors are highlighted to illustrate both the opportunities and the practical constraints of current technologies.

## 2. Low-Temperature Sintering Inks

The synthesis and formulation of high-performance functional inks require a strategic integration of material science and fluid dynamics. Initial design begins with material selection, where active phases, including metal, metal oxide, metal chalcogenide, and other specialized materials, are chosen to achieve specific electronic functionalities at temperatures compatible with heat-sensitive substrates. Beyond material selection, the rheological properties of inks, such as viscosity and surface tension play a critical role in determining the compatible printing methods and suitable substrates. For example, inkjet printing requires low (1–20 mPa·s) ink viscosities to avoid problems with droplet formation during printing, while EHD printing may benefit from inks with higher viscosity (100–1000 mPa·s) [76,77,78]. Surface tension and wettability effects must also be considered to fabricate consistent lines or different patterns for high resolution and optimal device performance [79,80]. Other parameters, such as electrical capabilities, mechanical properties, and costs, depend on the primary ink material. In the following section, we discuss different low-temperature sintering ink platforms based on their distinct material choices.

### 2.1. Metal Inks

Metal inks are the most widely used materials in various kinds of electronic devices, largely due to their extraordinary thermal and electrical conductivities. Among the diverse range of available metals, silver (Ag) and copper (Cu) serve as the two primary conductive materials in modern electrical engineering. Ag has the highest conductivity, regardless of its significant price volatility and rising costs. In contrast, Cu possesses slightly lower conductivity but is far more earth-abundant and cost-effective. While the current expansion of data centers has driven Cu prices to historic highs, it remains a substantially cheaper alternative to Ag. Beyond cost, Cu offers distinct technical advantages, most notably superior resistance to electromigration compared to Ag, which is critical for long-term reliability in high-current-density microelectronics. Despite their high performance, both Ag and Cu suffer from chemical instability, such as oxidation and atmospheric corrosion, which can compromise their functionality in high-temperature or corrosive environments. This necessitates the use of noble metals for specialized applications. Gold (Au), though less conductive than Ag or Cu, is highly chemically inert. This ensures a clean, reliable, and non-oxidizing connection over many years, particularly under harsh conditions such as high temperature and high humidity. In addition, Au is biocompatible and exhibits minimal electron leakage, enabling highly reliable and low-noise signal transduction in biosensor applications [81]. Furthermore, other metals such as nickel (Ni) and platinum (Pt) are used as alternative conductive metals for some specific applications. Ultimately, the selection of a specific metal ink for printed devices requires optimization of electrical performance, material cost, and environmental durability. Table 1 summarizes composition, sintering temperature, resistivity, and key features for metal inks. Particle-based inks for metals are well documented in the literature and have been the choice for printed electronics for many years. However, low-temperature sintering particle-free inks are increasingly preferred over traditional nanoparticle-based inks due to their superior shelf-stability, reduced risk of nozzle clogging, and their ability to achieve high-purity metallic films at significantly lower processing temperatures.

(1) Ag inks

Among the materials used for metal-based inks, silver (Ag) is the most common due to its excellent conductivity. Traditionally, Ag-based inks are composed of nanoparticles suspended in aqueous or organic-based solvents [78,87,88,107]. Within this platform, Ag nanoparticles are stabilized using a dispersing agent that prevents particle agglomeration and settling. Nanosized metal particles are preferred for their reduced melting temperatures that can be taken advantage of through reduced sintering temperatures [108]. An important component of nanoparticle inks is the choice of dispersing agent. Strong dispersing agents can enhance the colloidal stability of metal nanoparticles, yet they can also form an interfacial layer that disrupts electron transport upon ink drying [109]. Therefore, depending on the dispersing agent used, moderately high sintering temps (>200 °C) are used to thermally decompose the dispersing agent to avoid negatively impacting the conductivity of printed lines. Low-sintering-temperature inks are critical for biosensor applications, due to the low thermal resistance of substrates used to create devices that conform to the body [12].

**Figure 2:**
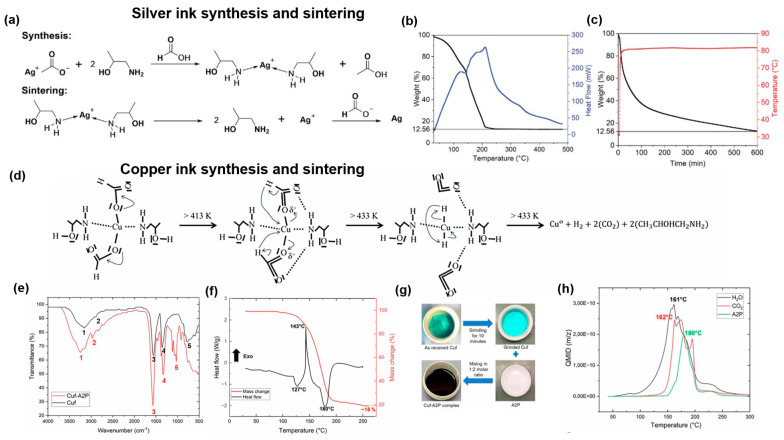
(**a**) Synthesis and decomposition reactions of the Ag-AP-based MOD precursor. (**b**,**c**) Thermogravimetric analysis of the ink under nitrogen at varying temperatures and durations. Reproduced with permission [92]. (**d**) Decomposition reaction mechanism of Cuf-A2P amine complex. (**e**) Cuf-A2P complex (red) investigated for various chemical bonds present in it compared to Cuf (black) using FTIR. (**f**) DSC (black) and TGA (red) analysis of Cuf-A2P, indicating various endothermic and exothermic reactions along with the mass loss taking place during the thermal decomposition of the Cu complex. (**g**) Synthesis of Cu-A2P complex. (**h**) Corresponding mass spectrum of Cuf-A2P complex indicating the release of gaseous byproducts during the thermal decomposition. Reproduced with permission [93].

Particle-free reactive inks have garnered attention due to their potential applications requiring low sintering temperatures. Unlike nanoparticle-based inks, reactive inks consist of metal precursors such as metal organic complexes or salts dissolved in a solvent with no stabilizers [53,110,111]. After printing, the precursors undergo a degradation process leading to the formation of highly conductive metallic films at much lower sintering temperatures (<120 °C). In one study, reactive Ag MOD inks were developed by first creating a solid Ag complex, μ-oxolato-bis(ethylenediaminesilver(I), that could be dissolved into an organic solvent mixture [91]. The printed ink patterns were thermally and photonically cured at 90 °C and 120 °C, respectively, creating uniform films on both PET and polyimide substrates. The resistivity of the Ag prints reached approximately 11 times that of bulk Ag. In another study, the sintering temperature was further reduced to room temperature through the synthesis of a novel diamminesilver(I) complex, facilitated by the alkanolamine 1-aminopropan-2-ol (AP) (Figure 2a–c) [92]. Upon solvent evaporation, the Ag complex readily decomposes into metallic Ag due to the high reactivity of the complex, creating highly conductive circuits on temperature-sensitive substrates.

To further lower the sintering temperature, chemical sintering methods have been investigated. One study developed Ag ink capable of self-sintering at room temperature, stabilized using polyacrylic acid sodium salt (PAA-NA) [82]. By investigating the adsorption mechanism of PAA-NA on the Ag nanoparticles, the desorption of the dispersing agent could be controlled after printing by treatment with chloride ions, destabilizing the particles and promoting coalescence. This chemical sintering, completed at room temperature, can achieve conductivities within 10% of bulk Ag.

Bulk Ag flakes have been another important component in Ag inks for printing. Commercially available Ag-flake-based inks are widely used as 3D printable conductive inks because micron-scale Ag flakes (typically 1–10 µm) provide high electrical conductivity through percolated metallic networks. The inks are typically formulated by dispersing Ag flakes in a polymer binder (e.g., epoxy, polyurethane, or elastomers such as PDMS) with suitable solvents and rheology modifiers to obtain high-viscosity, shear-thinning pastes compatible with additive manufacturing [112,113]. These inks can be patterned using extrusion-based 3D printing techniques such as screen printing, direct ink writing (DIW), robocasting, or aerosol-assisted printing, enabling the fabrication of conductive interconnects, electrodes, and multilayer structures [112,113]. After printing, the structures are thermally or photothermally sintered (80–250 °C) to remove solvents and partially decompose the binder, allowing close contact and neck formation between Ag flakes that significantly reduces electrical resistance. Alternative sintering approaches, such as photonic, laser, or microwave sintering, are also used to achieve rapid conductivity development while maintaining compatibility with temperature-sensitive substrates.

(2) Cu inks

Low-temperature sintering Cu ink, specifically Cu MOD ink, offers a significant advantage over traditional particle-based ink. It effectively overcomes the issue of Cu oxidation during storage due to the superior chemical stability of Cu^2+^ ions within the precursor complex. Over the past few decades, a number of Cu salts have been studied as the precursor for the formulation of Cu MOD inks, such as Cu sulfate [114], Cu nitrate [115], and Cu formate (Cuf). Among these, Cuf has dominated the research on Cu MOD ink development due to several unique advantages. First, Cuf is self-reducible, eliminating the need for an external reducing atmosphere to convert Cu ions into metallic Cu upon decomposition. This is because the decomposition of formate ions produces byproducts, including hydrogen gas, carbon dioxide, and formic acid, which creates an in situ reducing atmosphere. Second, the decomposition yields metallic Cu with minimal residuals, as the byproducts are volatile and easily eliminated. This ensures high purity and high conductivity in the printed tracks. In addition, Cuf possesses shorter anions compared to counterparts with similar coordination environments, enabling high Cu loading within the MOD inks.

The decomposition of Cuf follows either a one-step or two-step weight loss process, depending on whether the salt is hydrated [116,117]. In the case of Cuf tetrahydrate, which is the common commercially available salt without any further treatment, the initial weight loss upon decomposition is attributed to the dehydration of tetrahydrate in the crystalline structure. Thermogravimetric analysis reveals three endothermic peaks at 52, 80, and 102 °C, corresponding to the sequential removal of the first, second, and third water molecules of hydration, respectively. This process converts Cuf tetrahydrate into monohydrate, eliminating approximately 27% water from the precursor. Further decomposition starts slightly below 200 °C, releasing hydrogen gas, carbon dioxide, and water, with a characteristic exothermic peak at 217 °C [118]. The reaction typically completes by 250 °C [118]. In contrast, anhydrous Cuf possesses a higher Cu content and bypasses the low-temperature dehydration stage. Its decomposition into metallic Cu follows a similar mechanism, with the most dramatic reaction occurring between 200 and 250 °C. However, the thermogravimetric analysis in air indicates that the resulting metallic Cu undergoes rapid oxidation below 250 °C, leading to the formation of Cu_2_O and CuO. This presents an intrinsic challenge for sintering, as these oxides are electrically insulating and significantly detrimental to the conductivity of the printed Cu tracks.

It is worth noting that the decomposition of Cuf into metallic Cu still requires temperatures exceeding 200 °C, which serves as a critical threshold for many low-temperature sintering applications. Although advanced techniques such as laser and photonic sintering can be utilized as alternatives to thermal heating to prevent high-temperature damage, these methods rely heavily on specialized and expensive equipment. To tackle this challenge, there have been extensive studies investigating ink formulations. Complexing agents, such as amines, have been found to effectively reduce the sintering temperature of Cuf. As these amines possess lower boiling or decomposition temperatures (<200 °C), the sintering process still releases all gaseous byproducts, ensuring that high-purity Cu is left behind. In the case of alkylamines, such as ethylamine, propylamine, and butylamine, the complexation between the amine and Cuf significantly lowers the decomposition temperature. Under a N_2_ atmosphere, it was found that the final decomposition temperature decreased by 45–75 °C, making it possible to obtain fully metallic Cu below 200 °C [94]. Further investigation into the decomposition mechanism indicated that Cuf–amine complexes undergo a series of endothermic and exothermic processes, distinct from the single exothermic process observed for pure Cuf. The first stage occurs between 100 and 120 °C, where the carboxyl group dissociates from the Cuf first, leading to the reduction of Cu(II). The dissociation of the amine occurs next as the temperature rises, marking the second stage (endothermic). During this phase, Cu reduction is completed, and amines are detected via FTIR. When a longer alkylamine is used, the decomposition process tends to conclude at a higher temperature; for example, the Cuf-octylamine complex involves a third stage at 175 °C, where the undecomposed amine is finally released. When the decomposition occurs in air. Dong et al. observed that metallic Cu produced upon the decomposition of Cu-butylamine complex at the first stage (from 118 to 150 °C) quickly oxidized to Cu_2_O, while temperatures above 250 °C resulted in further oxidation and the formation of CuO [118].

Since amines as complexing agents not only affect the decomposition of Cuf but also contribute to the sintering of Cu particles, extensive efforts have been made to fine-tune the structure, coordination group, and amine-to-metal ratio to optimize Cu conductivity. In one study, monoalkylamines with varying chain lengths were systematically screened, revealing a synergistic effect when amines containing both long and short alkyl chains were employed. The replacement of monoamines by diamines was reported to promote the densification of the Cu film and prevent the Cu oxidation by consuming absorbed oxygen during the heating process [118]. By investigating various primary, secondary, and pyridine ligands, Paquet et al. established a rationale for producing highly conductive Cu films. The optimal amines (e.g., 2-ethyl-1-hexylamine, octylamines, and decylamine) possess strong coordination capabilities and intermediate boiling points. These properties stabilize small particle growth and prevent porosity or cracking, while still allowing the ligands to desorb completely once Cu reduction is finished [95]. Alkanolamines are also promising complexing ligands [96]. The presence of hydroxyl groups enables good solubility of the Cu complexes in many alcohol-based solvents, thereby extending the available solvent systems for printing. Moreover, these ligands bind in a bidentate fashion to the Cu(II) species, providing an improved oxidation barrier that protects Cu during sintering under ambient conditions [97]. With the assistance of 1-amino-2-propanol (A2P), a Cu MOD ink achieved low-temperature sintering while exhibiting optimal fluid properties (Figure 2d–h) [93]. The decomposition proceeds through dehydration at 60 °C, decarboxylation and Cu reduction at 143 °C, and final amine evaporation by 180 °C. By diluting the complex with ethanol and stabilizers, the viscosity and surface tension of inks were refined to meet inkjet printing requirements for high-resolution traces on flexible substrates. Additional insights into ink design were gained by examining the influence on the decomposition profile of Cu precursor [39]. It was demonstrated that increased molecular strain in distorted 5-coordinate geometries, which were induced by sterically bulky alkanolamine ligands, decreases complex stability, favoring a lower decomposition temperature. By utilizing this rationale, inks can be processed at temperatures as low as 125–150 °C, enabling the use of heat-sensitive flexible substrates. A study further demonstrated that transitioning from linear to branched alkanolamine ligands optimizes Cu MOD inks by increasing solubility and inducing steric hindrance that lowers decomposition temperatures, resulting in Cu films with superior morphology and high conductivity [98]. Mixed ligand strategies have also been applied to alkanolamine ligands to further improve the performance of Cu inks. For instance, the addition of tetramethylethylenediamine improved the solubility of Cu-alkanolamine complex in water [99]. Additionally, the blending of bidentate 2-amino-2-methyl-1-propanol (AMP) with tridentate serinol was reported to create a synergistic effect that enhances both the solubility and the atmospheric stability of the Cu ink. This multidentate coordination provides a multiscale protection mechanism that suppresses oxidative aggregation during storage and creates a protective organic barrier during sintering to ensure high conductivity in air [100].

Additives also play an essential role in defining the properties of Cu MOD ink. Cellulose-based polymers, such as hydroxypropyl methylcellulose, are common rheological modifiers used to modulate ink viscosity and printability. Organic acids, including hexanoic acid and dodecanoic acid, have been found to act as sintering agents to promote the percolation and connectivity of the Cu prints [99,119]. Furthermore, stabilizing agents like PEG can improve the homogeneity and surface morphology of the sintered film [116]. Regarding the solvent system, a study suggested that an ideal solvent should possess a high boiling point, strong dissolving power for Cu salt, and chemical inactivity toward the formate anion to prevent interference with Cu reduction [120]. Nevertheless, as most Cu MOD inks still require a protective atmosphere (such as Ar or N_2_) or a vacuum environment for sintering, extensive research into innovative ink formulations remains critical. Fully addressing these ongoing oxidation challenges is essential to enabling robust, reliable processing in ambient air, which would significantly simplify manufacturing and reduce operational costs.

(3) Au, Ni, and other metal inks

In addition to Ag and Cu, other metal salts have been applied to formulate particle-free inks. Au is of significant interest for bio-related applications due to its excellent biocompatibility, high electrical conductivity, and inherent oxidation resistance. Metal–organic Au carboxylates have been utilized to form Au MOD ink, which yielded metallic Au at 150 °C. However, subsequent sintering at 280 °C is required to achieve maximum conductivity [101]. HAuCl_4_ is an excellent Au precursor due to its high reduction potential. These inks can be converted to Au at room temperature using plasma [102], laser [103], or UV light irradiation [104], through a photochemical reduction process. Ni inks have also drawn some interest because of the excellent conductivity, high magnetic permeability, and good oxidation resistance. The chemistry for ink formulation is often based on Ni formate-amine complexes. However, the complete conversion of Ni complex to elemental Ni typically requires sintering temperatures exceeding 200 °C [105]. To address this, Li et al. proposed a dual-ligand strategy to coordinate Ni^2+^, where a combination of σ-donor and π-donor amine ligands effectively lifts the orbital degeneracy of the Ni^2+^ center, enhances electron transfer, and lowers the pyrolysis temperature to 180 °C [106]. Unlike MOD inks that require complexing agents to stabilize metal salts, it was demonstrated that inorganic metal salts can be dissolved in a mixture of water and ethylene glycol. Due to their sufficiently positive reduction potentials, these metal salts can be readily reduced to elemental metals, including Au, Pd, Pt, Bi, Pb, and Sn, via a plasma-triggered reduction process [121,122]. Furthermore, inks composed of a mixture of metal precursors with adjusted stoichiometry can facilitate the formation of alloys. In these systems, ink chemistry is critical. The precursors must dissolve in the same carrier vehicle and decompose at similar post-processing temperatures. The metals must be chemically compatible to ensure a homogeneous phase rather than undesirable phase separation. For example, metal acetates and carbonyls have been used to form the Pd-Ag [123] and Fe-Co [124] MOD inks, respectively. Similarly, a combination of Cu and Ni MOD inks can result in the formation of a Cu-Ni alloy [125] or distinctive structural characteristics, such as a core–shell configuration with enhanced oxidation resistance [98].

### 2.2. Metal Oxide Inks

Metal oxides (for example, SiO_2_, TiO_2_, ZnO, In_2_O_3_, SnO_2_, BaTiO_3,_ and NiO) are the backbone of printed electronics, offering additional functionalities beyond conductivity [10,126,127,128,129]. Although conventional vapor phase deposition produces high-quality metal oxide films, it requires vacuum systems, elevated temperatures, and complex processing, which present inherent challenges for macro-scale device fabrication. In contrast, solution processing provides a low-cost and scalable route for printed electronic device manufacturing. However, despite these advantages, solution-processed metal oxides often contain higher levels of impurities and defects, particularly residual organic species, which remain a critical bottleneck for their application in printed electronics. The conversion of precursor inks into dense, low-defect oxide networks typically requires annealing temperatures exceeding 500 °C, incompatible with printed electronics, which are often fabricated on polymer substrates. Such high temperatures also make the use of metal oxide ceramic particles in inks impractical. Consequently, extensive efforts have focused on low-temperature sintering strategies (<200 °C) that preserve film quality while minimizing thermal load. One way to address this limitation is to replace particle-based ink with a precursor system. Unlike nanoparticle inks where sintering is governed by particle coalescence, precursor inks require chemically driven transformations, such as hydrolysis-condensation, nitrate decomposition, and ligand removal. These reactions enable the formation of the final oxide network at significantly lower temperatures than conventional sintering. Here, we explain the chemistry of metal oxide precursor inks, including sol-gel, metal alkoxide systems, metal nitrate systems, and combustion-based formulations, together with low-temperature activation pathways such as deep UV irradiation, photonic curing, oxidative treatments, microwave processing, and infrared annealing.

(1) Sol-gel condensation

**Figure 3:**
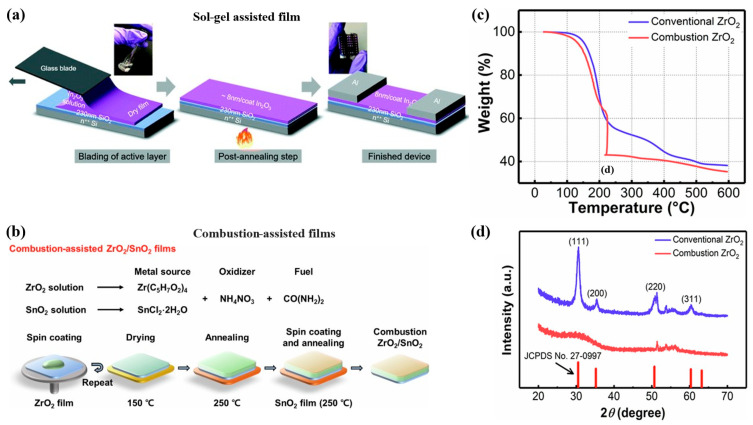
(**a**) Schematic representation of the various stages involved in blade-coating of In_2_O_3_ by sol-gel method. Reproduced with permission [135]. (**b**) Schematic of ZrO_2_/SnO_2_ films using combustion solutions; (**c**) TGA spectra of ZrO_2_ precursors with and without combustion materials and (**d**) GIXRD spectra of ZrO_2_ films prepared from different precursors. Reproduced with permission [144].

The sol-gel process is a wet-chemical technique mostly used for the fabrication of metal oxide ceramic materials. In this process, the sol (or solution) evolves gradually towards the formation of a gel-like network containing both a liquid phase and a solid phase. Typical precursors are metal alkoxides and metal chlorides, which undergo hydrolysis and polycondensation reactions to form a colloid. This route creates an intermediate involving a metal-amine and metal alkoxide complex, which transforms into a metal hydroxide species under acidic or basic hydrolysis. Conversion to a metal oxide is completed by heat treatment at 200–300 °C, forming the final M–O–M network from an M–OH network [130,131,132,133]. The Stöber process is a well-known sol-gel approach to prepare the dielectric SiO_2_ thin film, and it was utilized as a gate dielectric in thin-film transistors [129,134]. Sol-gel-derived inks are widely used due to their compositional tunability and high film uniformity. However, incomplete ligand removal and residual hydroxyl species at low temperatures lead to trap states and degraded electrical performance. Kirmani et al. demonstrated that sol-gel and combustion chemistries can produce amorphous In_2_O_3_ at temperatures as low as 200 to 225 °C (Figure 3a) [135]. Heat treatment at around 300 °C can completely remove the precursor species and fully convert the metal hydroxide into metal oxide [127,136]. In another example, Banger et al. utilized a “sol-gel on chip” hydrolysis approach to fabricate amorphous IZO (In*_x_*^1^Zn*_y_*^2^O*_z_*) based on metal alkoxide precursors [126]. It is significant that this method produces metal oxide at low temperature (230 °C) through direct hydrolysis reaction using metal alkoxide precursors without intermediated hydrolysis reaction. If a lower temperature is employed, ~200 °C, there will be some -OH species left behind. These films can be converted by a photochemical route using UV exposure. This cleaves the O-H bond in M-OH and converts it to the networked M–O–M as well as oxidizing residual hydroxyl and organic species [135,137,138]. Kim et al. developed a new photo-annealing method for forming amorphous metal oxides and examined its viability for producing high-quality metal oxide layers on polymer substrates [139]. They use photochemical activation induced by deep-ultraviolet (DUV) light from a low-pressure mercury lamp in an inert atmosphere (to prevent reactive ozone formation) to achieve high degrees of sol-gel condensation and film densification in systems including indium gallium zinc oxide (IGZO), indium zinc oxide (IZO), and indium oxide (In_2_O_3_) [139]. Deep-ultraviolet (DUV) irradiation [140] and UV–ozone treatments [141] have also emerged as effective low-temperature activation routes, enabling photolytic cleavage of organic ligands and accelerated M–O–M network formation.

(2) Combustion-assisted oxide formation

Another method to avoid higher temperatures for sintering is to use combustion-assisted reactions to form the metal oxide film [128]. When a combustion reaction takes place within the deposited ink film, it provides a more localized heat source [142,143]. This allows the precursor to convert to the final metal oxide at a reduced overall sintering temperature. Two precursors are required for this reaction: (i) an oxidizer and (ii) a fuel source [128,144]. The oxidizer is often a metal nitrate and metal halide, while the fuel can span a broader range of compounds. Common fuel precursors include urea [144] and citric acid [142,145]. Additionally, sometimes the solvent itself can act as the fuel source, such as in the case of 2-methoxyethanol [142]. In a typical system for zinc, indium, or mixed-metal oxides, the metal nitrates supply both the metal cations and the oxidizing power [132,142,145]. As with sol-gel condensation, a thin film is important for combustion-assisted sintering because of the volatile compounds that are produced and must escape during the reaction [142,143,145]. Jang et al. demonstrated this approach using SnO_2_ and high-k ZrO_2_ materials, where flexible substrates were used for fabrication (Figure 3b–d) [144]. It showed excellent mechanical stability, capable of withstanding 5000 cycles of bending tests at a bending radius of 2.5 m. The SnO_2_ semiconductors and high-k ZrO_2_ materials are both synthesized through combustion-assisted sol-gel processes [144]. This method involved the exothermic reaction of urea as fuel and ammonium nitrate as oxidizer to produce high-quality oxide films without extensive external heating [144]. The combustion ZrO_2_ films were revealed to have an amorphous structure with a higher proportion of oxygen corresponding to the oxide network, which contributes to the dielectric properties [144].

(3) Peroxides and nitrates

Metal nitrate precursors offer a distinct advantage through their intrinsic oxidizing nature, which can undergo self-decomposition to oxide formation. Peroxo–metal complexes are also used with the metal precursor to provide enhanced oxidizing power during decomposition to the metal oxide. Both metal nitrate and peroxides can undergo self-decomposition and redox reactions, but most often, they are enhanced by fuel additives (e.g., urea, acetylacetone), leading to combustion-assisted oxide formation [135]. This allows for low temperatures of approximately 200–250 °C to be used, producing dense metal oxide layers [146,147]. A related strategy is to use carefully designed metal nitrate precursor systems [132,148]. These systems are governed by the hydration state of the precursor, with a lower hydration state allowing breakdown to occur at a lower temperature. The use of nitrates as a precursor and the exclusion of a fuel source allow for the formation of amorphous films with a cleaner final product [148,149]. Additionally, by controlling the hydration state, a greater level of control can be achieved [148,150,151,152].

### 2.3. Metal Chalcogenide Inks

Metal chalcogenides (sulfides, selenides, and tellurides) are semiconductor materials that exhibit size-dependent optical and electrical properties. When their dimensions are reduced to the nanoscale, at or below the exciton Bohr radius, these materials are referred to as quantum dots (QDs), a rapidly expanding class of functional nanomaterials [153,154,155]. Metal chalcogenide inks are typically processed via two main approaches: (i) reactive conversion routes, in which chalcogenidometallate precursor inks are printed and subsequently sintered to form the chalcogenide phase; and (ii) printing of preformed metal chalcogenide nanocrystals (NCs) or nanowires, where charge transport is achieved through percolation with minimal additional densification.

Among the different classes of QDs and NCs, lead sulfide (PbS)-based ones have particularly captured the attention due to their tunable absorption edge from the near-infrared through the visible region [156,157]. High-quality PbS NCs inks are mostly prepared by using the hot injection method (temperature 70–200 °C), followed by a layer-by-layer film deposition by ligand exchange to small molecules like ethane dithiol (EDT) or mercapto propanoic acid (MPA) [157,158,159]. Recent progress showed one-step ligand exchange with organic–inorganic capping, like perovskite, was also possible [160]. More recent progress showed direct synthesis of PbS and PbSe QD inks at room temperature [161,162]. In comparison to the conventional three-step synthesis, direct synthesis strategy simplifies the fabrication process to one step and reduces the preparation cost by a factor of eight.

**Figure 4:**
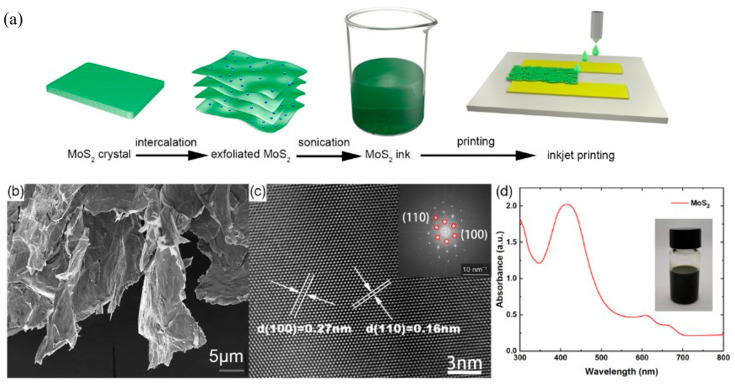
Metal chalcogenide inks. (**a**) Schematic of electrochemically exfoliated MoS_2_ ink for inkjet printing. (**b**,**c**) Electron microscopic image and (**d**) absorbance of MoS_2_ ink. Reproduced with permission [169].

Another interesting class of metal chalcogenides is 2D transition metal dichalcogenides, which are usually printed by following two distinct approaches: (1) printing of precursor inks followed by in situ conversion and (2) printing of pre-made 2D materials (exfoliated inks). The most common precursor is ammonium thiomolybdate (NH_4_)_2_MoS_4_) for making the precursor ink; unfortunately, it requires a very high sintering temperature (800–1000 °C) [163,164]. Molybdenum dithiocarbamate and molybdenum dithiocarboxylate are well-known precursors that have previously been used to prepare MoS_2_ thin films comparatively at lower annealing temperatures (450–500 °C) by aerosol-assisted chemical vapor deposition [165,166], and these precursor-based inks could be leveraged in the future for low-temperature MoS_2_ fabrication using different printing technology. Other approaches associated with pre-made 2D material inks rely on top-down synthesis routes, where bulk 2D materials are exfoliated into nanosheets first and subsequently formulated into printable dispersions. A recent study demonstrated a temperature-controlled exfoliation strategy in which multiple 2D materials, including graphene and 2D MoS_2_, were directly dispersed in pure water without surfactants, achieving high stability (over a month) and compatibility with printing processes [167]. A broad review of solutions processed from pre-made 2D materials highlights how assembly/film formation governs electronic/optoelectronic performance, frequently within low thermal budgets compatible with flexible substrates [168]. Beyond mechanical exfoliation, electrochemical exfoliation is a well-established technique. In one study, MoS_2_ ink was synthesized by electrochemical exfoliation of bulk MoS_2_ using quaternary ammonium ion intercalation, followed by sonication and redispersion of few-layer (2H-phase) nanosheets in a mixed solvent system to achieve printable viscosity and surface tension (Figure 4) [169]. The resulting stable ink was inkjet-printed to form uniform MoS_2_ films, which were post-treated to reduce defects and improve charge transport [169].

### 2.4. Nanoparticle Inks Synthesized with Biobased Polymers

Beyond precursor-based ink, particle-based metal or other nanoparticle inks are also important for printed electronics using low-temperature sintering inks. Metal nanoparticles have mainly two constituents: the inorganic metal as core and the organic molecule as shell. The outer organic molecule as shell is required to protect/cap the nanoparticles. The outer organic molecule could be an alkyl amine, alcohol, carboxylic acid, or polymeric component. Using polymeric components as a capping agent has some rheological advantages for the printing process, as these polymers may act as thickening agents, steric stabilizers, or capping agents that prevent nanoparticle aggregation. Polyvinylpyrrolidone (PVP) is most widely used in commercial ink formulations because it is biocompatible, non-toxic, and easy to process [89,90]. However, PVP is a synthetic polymer derived from petrochemical feedstocks, raising sustainability and cost concerns for large-scale production.

Recent advances in cellulose chemistry have established cellulose-based polymers as promising alternatives to PVP for ink formulation. Cellulose derivatives such as hydroxyethyl cellulose (HEC) are non-toxic, renewable, and biobased, offering clear environmental and economic advantages. Beyond their role as rheology modifiers, cellulose polymers can also function as capping agents. HEC contains abundant hydroxyl groups capable of donating and accepting electrons, enabling coordination with Ag ions during nanoparticle synthesis. This dual functionality allows HEC to control particle growth while simultaneously stabilizing the dispersion. Although cellulose materials have traditionally been used only as post-added rheology modifiers, recent work demonstrated that HEC can serve as an effective capping agent for EHD printable Ag nanoparticle inks with exceptional long-term stability extending over several months [9,78].

In this system, nanoparticle size was controlled through two independent mechanisms: the AgNO_3_-to-HEC weight ratio and solution pH [78]. Importantly, the lower thermal decomposition temperature of HEC compared with PVP translated into substantially reduced sintering temperatures [9]. Conductive films with resistivities as low as 13.38 µΩ·cm were achieved after sintering at 150 °C for 30 min, whereas commercial PVP-based inks required temperatures exceeding 350 °C to reach comparable conductivities. This reduction in sintering temperature enables printing on a wider range of flexible polymer substrates, lowers energy consumption during manufacturing, and increases production throughput. The robustness of this HEC-based ink was further demonstrated by successful printing under microgravity conditions during parabolic flight, highlighting its suitability for EHD printing in unconventional environments [78].

Further insight into the role of nanoparticle size was obtained using HEC as a capping agent decoupled from size control [9]. In conventional PVP-based systems, increasing polymer concentration reduces particle size, making it difficult to isolate size effects on conductivity. By contrast, HEC enabled independent size control through pH adjustment while maintaining constant polymer concentration. Surprisingly, smaller Ag nanoparticles produced higher conductivity than larger counterparts, contradicting earlier reports that favored larger particles. This behavior was attributed to improved packing density and interparticle contact enabled by uniform surface chemistry. In addition to electrical performance, particle size control using HEC also led to enhanced mechanical robustness of printed films [9].

Overall, cellulose-based polymers, particularly HEC, offer a sustainable, low-temperature, and structurally versatile alternative to PVP for conductive ink formulation. Their ability to reduce sintering temperatures, enable unique nanostructure growth, and decouple particle size from polymer concentration provides new design freedom for printed electronics. These advances support the development of high-performance conductive inks compatible with flexible substrates, energy-efficient manufacturing, and next-generation printing platforms.

### 2.5. Conductive Polymer Inks

Conducting polymers are organic macromolecules featuring conjugated π-electron systems that exhibit electrical conductivity ranging from semiconductor to metal-like levels. These flexible, lightweight materials are generally produced through chemical or electrochemical polymerization and require doping to enhance conductivity, making them valuable for sensors, flexible electronics, energy storage, and corrosion protection. PEDOT:PSS is most widely used as a 3D-printable conductive ink because it is water-processable, biocompatible, and supports mixed ionic/electronic transport, which is advantageous for bio-interfaces and biosensor transduction. To achieve extrusion/DIW printability, PEDOT:PSS is commonly converted into viscoelastic, yield-stress inks via approaches such as ionic-liquid–facilitated colloidal stacking (enabling tall 3D structures with high conductivity and “one-shot” biocompatibility) [22] or deep-eutectic-solvent eutectic-gels that print substrate-free, conformal dry electrodes for electrophysiology (e.g., EMG) [170]. Beyond DIW, PEDOT-based formulations can also be adapted to DLP/photopolymerization 3D printing to form shape-defined conductive hydrogels and patterned electrodes with controlled geometries [171], and these material/processing concepts are increasingly used to fabricate fully printed bioelectronic components such as 3D-printed OECT architectures for sensing and interfacing [172].

### 2.6. Emerging Material Inks

Beyond conventional metallic systems, emerging material inks such as liquid metals, perovskites, and framework-based materials offer new pathways for low-temperature processing and device integration. Liquid metal (LM) inks are excellent conductive ink candidates beyond traditional metal inks as they offer a combination of high electrical conductivity and fluidic deformability. These inks can have exceptional structural adaptability to form 3D or biological surfaces even under room temperature. A common LM material is eutectic gallium-indium (EGaIn), which remains in a liquid state at room temperature, allowing it to be directly utilized as functional inks without intensive thermal processing [173,174]. For example, a gallium-based ink was developed for variable stiffness electronics; in this case, a pH-controlled sintering process was used to remove oxide layers and interconnect metal particles, improving the mechanical tunability and conductivity [175].

Metal–organic frameworks (MOFs) and covalent organic frameworks (COFs) are highly versatile, 3D nanoporous materials. Their exceptional properties, such as high surface area, tunable porosity, and chemical adaptability, make them ideal candidates for sensing, electronics, and membrane applications. These inks generally contain the framework particles or their molecular precursors as the active phase to provide the required functional properties. Similar to other functional inks, solvents, surfactants, binders, and rheological modifiers are carefully formulated to enhance colloidal stability, printability, and substrate adhesion [176]. The sintering process for these materials is distinct, as it focuses on low-temperature “activation” or in situ crystallization without causing the thermal collapse of the organic framework [177].

## 3. Low-Temperature Sintering/Curing Process and Mechanism

The sintering mechanism dictates the final microstructure and electrical efficacy. While high-energy methods like photonic sintering offer speed, steady-state thermal curing remains the benchmark for uniform electrical performance [178,179]. Furthermore, emerging “cold sintering” processes (CSP) provide a pathway for densification through transient liquid phases at very low temperatures [180]. Low-temperature sintering methods, their advantages, and limitations are summarized in Table 2.

### 3.1. Thermal Curing (<200 °C): Convection/Hotplate/IR

**Figure 5:**
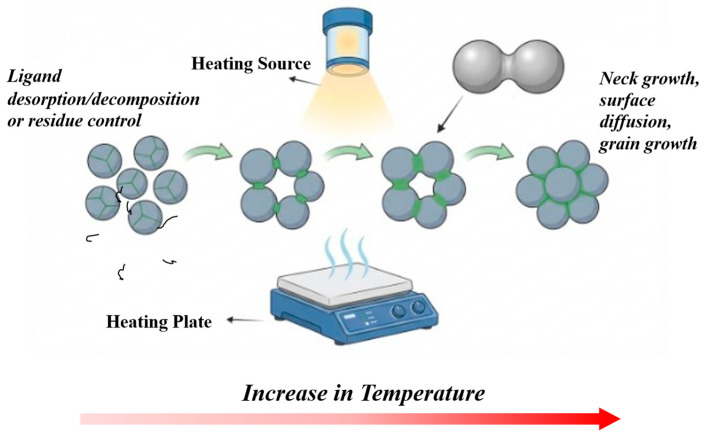
Low-temperature sintering process and mechanism. Adapted from reference [179].

Thermal curing provides the activation energy required for metallic consolidation while managing the competition between solvent evaporation and organic removal, as shown in Figure 5 [181,183].

(1) Densification

The densification of printed inks during thermal sintering is a complex physical process centered on the reduction of porosity to enhance structural and electrical integrity. At T < 200 °C, this process is governed by the Gibbs–Thomson effect, causing significant melting temperature (T_m_) depression as particle diameter decreases [179]. This thermodynamic shift facilitates early-stage neck growth [204]. For instance, in 50 nm Ag nanoparticles, surface diffusion initiates as low as 60 °C [179]. Atoms migrate from convex surfaces to contact points (necks). By 120 °C, substantial neck growth establishes the conductive pathways necessary to reduce film resistivity [179,204]. Conductivity follows a percolation model. Introducing dielectric modifications, such as SiO_2_/Ag nanoparticles, allows for the optimization of mechanical flexibility without disrupting the conductive network [205]. While thermal curing often leaves residual porosity, optimized packing and thermal input can yield conductivities of 30–40% of bulk Ag [179,182].

(2) Organic removal: ligand desorption and residue control

Insulating organic shells (e.g., PVP) are the primary barriers to connectivity. To overcome this, thermal energy is employed to trigger ligand desorption. Modern inks utilize ligands with lower binding energies to facilitate removal at 120–150 °C [179,182]. Unlike ultra-fast sintering techniques, steady-state thermal curing allows for more uniform organic removal than ultra-fast methods. However, the temperature ramp must be managed to prevent “skin” formation, which can trap carbonaceous residues and cause film cracking [182,183].

(3) Emerging conversion and densification pathways

Beyond traditional nanoparticle fusion, several emerging pathways offer sophisticated mechanisms for achieving film integrity at reduced thermal budgets. For example, MOD inks utilize complexes that decompose to form highly reactive metallic clusters in situ, enabling dense networks at temperatures as low as 100 °C [183]. Alternatively, solution-based sintering strategies employ reactive binders, such as VegPU, to facilitate an interfacial chemical reaction between Ag flakes. This yields exceptional stretchability (350%) and high conductivity (~12,833 S/cm) for soft electronic applications [178,181]. Finally, the cold sintering process (CSP) introduces a dissolution–precipitation mechanism that enables densification through a dissolution–precipitation mechanism at temperatures typically <300 °C. By utilizing a transient liquid phase and low uniaxial pressure, CSP can achieve near-theoretical densities for ceramics and composites, offering a unique pathway for co-processing metallic inks with inorganic functional layers [180].

By leveraging nanoparticle surface-diffusion kinetics and optimized precursor decomposition pathways, researchers have achieved significant metallic densification at temperatures below 200 °C. The integration of sustainable solution sintering, physiological phase-transition triggers [183], and cold sintering methodologies [178,180] provides a versatile roadmap for the next generation of bio-integrated and stretchable devices. Future advancements will likely focus on the synergistic combination of these steady-state thermal mechanisms with localized, high-speed photonic processing to facilitate the transition from laboratory prototypes to industrial-scale flexible manufacturing.

### 3.2. Photonic Sintering

Photonic sintering (PS) is a high-speed thermal treatment technique used to densify thin-film materials using light from xenon flash lamps [184,185]. The light from the lamps induces localized heating within the surface/layer, and because metallic nanoparticles exhibit high absorption cross-sections, this allows them to reach temperatures exceeding 500 °C within milliseconds [186]. However, energy coupling efficiency is highly dependent on the optical properties of the ink, and a material with a wide bandgap can be challenging to sinter using this method [184]. Because this energy is delivered extremely rapidly, atoms have sufficient mobility to diffuse across particle boundaries, eliminating pores and establishing a conductive percolating network. Rapid heating and cooling during photonic sintering can also introduce thermal stresses within the printed film, which may lead to cracking or delamination, particularly when there is a mismatch in thermal expansion coefficients between the conductive layer and the substrate. This effect becomes more pronounced for thicker films or inks containing rigid binders. Several studies have shown that these issues can be mitigated through optimization of pulse energy, pulse duration, and multi-pulse processing strategies, as well as through ink formulations that incorporate flexible polymer binders or graded structures that relieve mechanical stress. The rapid surface-induced heating limits the penetration depth of the energy pulses [187]. As a result, the bulk of the substrate remains near ambient temperature, thus avoiding the thermal degradation that would occur in a conventional sintering oven [188]. The speed at which energy is delivered to the surface allows PS to be used in high-speed sintering lines; this rapid energy delivery enables roll-to-roll processing by eliminating long curing times that would otherwise act as a manufacturing bottleneck. Despite these advantages, photonic sintering systems typically require specialized xenon flash-lamp equipment, which can involve relatively high initial capital costs compared with conventional thermal ovens. However, the ability to perform millisecond-scale sintering and compatibility with roll-to-roll manufacturing often offset these costs in industrial printed electronics production.

### 3.3. Laser Sintering

Another sintering method available is selective laser sintering (SLS). A scanning laser beam can be focused on specific patterns, which is known as spatial selectivity. This selectivity allows sintering of micro-patterns on flexible substrates [189]. Similar to photonic sintering, laser sintering can generate localized thermal gradients, which may introduce cracks, warping, or delamination in printed films, particularly when processing multilayer structures or inks containing high binder content. Careful control of laser power, scan speed, beam diameter, and pulse duration is, therefore, required to minimize thermal stress while still achieving sufficient particle necking and densification. This method shares some similarities with Powder Bed Fusion, as both utilize powder beds and laser arrays to selectively lase and melt (in PBF the powder is melted). This technique offers users the ability to manipulate beam shape, laser power, scan speed, and energy deposition rate on patterns and parts [190]. Like photonic sintering, most of the thermal energy is absorbed by the powder layer, and not the substrate, and the laser pulse duration is often shorter than the thermal relaxation time of the substrate (but this is dependent on the type of laser used) [190]. A continuous wave laser might have an interaction time longer than the thermal relaxation time, which creates a larger heat-affected zone [191]. This quick pulse duration allows substrates that would otherwise be destroyed in a traditional sintering oven to be used in the SLS process [191,192]. Laser sintering systems also generally involve higher equipment complexity and cost due to precision optics, scanning systems, and laser sources. While this may limit large-area industrial deployment compared with photonic sintering, the technique offers exceptional spatial resolution and selective patterning, making it particularly attractive for micro-scale printed electronics and sensor fabrication. However, in multilayer sintering, the laser does penetrate the powder stack and creates a heat-affected zone, which can inadvertently sinter adjacent particles [191,193].

### 3.4. Plasma and Microwave Sintering

Plasma and microwave flash sintering are both ultrafast processing techniques that densify ceramics in seconds [194]. Plasma sintering, as is typically integrated within a spark plasma sintering (SPS), uses a pulsed electric field applied to a powder compact, whereas microwave flash sintering relies on intense microwave radiation [195]. The mechanisms for densification are different between the two methods. The surface activation in plasma sintering is characterized by ionic stripping, in which high-energy plasma interacts with particle surfaces to remove oxide layers and adsorbed gases, effectively cleaning the surface, which promotes rapid mass transport and near-theoretical densities at lower macroscopic temperatures. In microwave sintering, the process is governed by a thermal runaway mechanism. Once a threshold temperature governed by an Arrhenius-type dependency is reached, the material’s ability to absorb microwave energy increases exponentially, which causes a self-reinforcing surge in internal Joule heating. This non-linear feedback loop leads to a flash event and a sudden rise in temperature and, in conjunction with defect generation, facilitates an instantaneous shrinkage event that is much faster than traditional diffusion-limited kinetics of conventional sintering furnaces [196].

### 3.5. Chemical Sintering

Chemical sintering is an alternative to conventional thermal or photonic sintering in which chemical reactions, rather than heat alone, drive interparticle contact formation, ligand removal, and oxide reduction in printed metallic films. This approach is particularly attractive for printed electronics on temperature-sensitive substrates because it can enable high conductivity at room temperature or mildly elevated temperatures (<150–200 °C). Chemical sintering is most often implemented through reactive solvents, acids, reducing agents, or halide and formate treatments that modify nanoparticle surfaces and promote metal–metal bonding.

For Ag nanoparticle inks, chemical sintering primarily involves removal or destabilization of organic capping ligands (e.g., PVP, amines) and enhancement of surface diffusion between particles. One widely used strategy is treatment with chloride-containing or acidic solutions, which screens steric repulsion and promote particles coalescence. For example, exposure of printed AgNP films at room temperature to cetyltrimethylammonium chloride (CTAC), NaCl, KCl, or HCl vapors/solutions induces rapid conductivity improvement to 20% of bulk Ag by disrupting polymeric stabilizers and enabling direct metal contact [82,83,84,85,197]. Another effective route uses formic acid or carboxylic acids, which partially dissolve or displace ligands and increase surface mobility of Ag atoms [86,198]. These methods can produce conductive Ag films at room temperature or below 100 °C, making them compatible with plastic and paper substrates. In particle-free or metal–organic decomposition (MOD) Ag inks, chemical sintering occurs via reduction of Ag complexes to metallic Ag, often assisted by alcohols, amines, or mild reducing agents [53].

Chemical sintering is even more important for Cu inks because Cu nanoparticles are readily oxidized and require simultaneous ligand removal and oxide reduction. A common strategy employs formic acid or formate salts, which act both as ligand-removal agents and as reducing agents for CuO/Cu_2_O. Treatment of printed CuNP films in formic acid vapor or solution can yield metallic Cu at temperatures as low as 100–150 °C under ambient or inert conditions [199,200,201]. Another widely explored route uses amine-based or hydrazine-based reducing systems, where chemical reduction of surface oxides enables neck formation between particles. In some systems, self-reducing inks are designed such that the solvent or ligand decomposes into reducing species during drying, eliminating the need for an external reducing atmosphere [96,202]. Overall, chemical sintering enables Cu and Ag films to be processed without high-temperature annealing or hydrogen atmospheres, which is critical for low-cost, roll-to-roll manufacturing on polymer substrates for printed electronics.

### 3.6. Trade-Offs Among Low-Temperature Sintering Approaches for Biosensor Fabrication

Different low-temperature sintering strategies present distinct advantages and limitations when applied to biosensor fabrication. Thermal curing remains the most widely used method because of its simplicity and uniform heating; however, processing times are typically longer, and temperatures above 120–150 °C may still degrade sensitive substrates or bio-functional layers. Photonic sintering enables millisecond-scale processing through selective light absorption by metallic nanoparticles, allowing the conductive layer to reach high temperatures while the substrate remains relatively cool. This approach is particularly attractive for roll-to-roll manufacturing of wearable biosensors but requires careful control of pulse energy to avoid cracking or delamination. Laser sintering provides high spatial resolution and enables selective activation of individual device regions, which is beneficial for micro-patterned biosensor electrodes. However, the technique requires precise control of laser parameters and typically involves higher equipment costs. Chemical sintering represents an alternative strategy that relies on surface chemical reactions to remove stabilizing ligands and promote interparticle bonding at room temperature or mildly elevated temperatures. This approach is particularly promising for biosensors because it minimizes thermal exposure and preserves the activity of biomolecular recognition elements. Microwave and plasma-assisted sintering provide rapid densification through volumetric or plasma-induced activation mechanisms. These techniques can significantly reduce processing times, but their compatibility with multilayer biosensor architecture remains an active area of research due to potential damage to underlying polymer substrates. Overall, the optimal sintering approach depends on the specific biosensor architecture, the thermal stability of the substrate and functional layers, and the desired manufacturing throughput.

From a biosensor-specific perspective, the suitability of different sintering approaches is determined not only by electrical performance but also by compatibility with bio-functional layers and device architecture. Thermal curing offers uniform densification but may induce degradation of enzymes or polymer substrates when exposure times are prolonged. Photonic and laser sintering provide localized energy delivery, enabling high conductivity while preserving temperature-sensitive substrates; however, rapid thermal gradients can lead to interfacial stress, cracking, or delamination in multilayer biosensor structures.

Chemical sintering presents a particularly attractive route for biosensors, as it enables room-temperature processing and minimizes damage to biological components. Nevertheless, it may introduce chemical residues that affect long-term stability or biocompatibility. Plasma and microwave-based methods offer rapid densification and oxide removal but remain limited by scalability and compatibility with complex multilayer architectures.

Overall, no single sintering approach is universally optimal; instead, the choice must be guided by the specific biosensor design, including substrate type, recognition chemistry, and required performance metrics such as sensitivity, response time, and long-term stability.

### 3.7. Impact of Sintering-Induced Microstructure on Biosensor Performance

Beyond achieving electrical conductivity, the microstructure formed during sintering plays a critical role in determining biosensor performance. The sintering process governs particle neck formation, grain growth, film porosity, and surface roughness, all of which influence electrochemical properties of the electrode interface [14,53,54]. Highly porous structures can increase electrochemically active surface area and improve analyte accessibility, enhancing sensitivity in catalytic or enzymatic biosensors [27,51]. However, excessive porosity may reduce electrical conductivity and introduce signal instability during long-term operation. Conversely, dense films provide excellent conductivity but may limit analyte diffusion to the electrode surface [35]. Therefore, optimizing sintering conditions requires balancing electrical conductivity, mechanical stability, and surface morphology to achieve reliable biosensor performance.

Different sintering methods for biosensor devices and their advantages and limitations are summarized in Table 3.

Different sintering strategies produce distinct microstructures that directly influence biosensor performance. Dense films produced by photonic or laser sintering typically provide low electrical resistance and stable signal transmission, whereas partially porous structures produced by thermal or chemical sintering can increase electrochemically active surface area, enhancing sensor sensitivity. Therefore, selecting a sintering approach requires balancing conductivity, surface morphology, and compatibility with biological functional layers.

Beyond substrate compatibility, sintering conditions directly influence biosensor performance by affecting electrode morphology, conductivity, and interfacial chemistry. For example, insufficient sintering can lead to porous or poorly connected conductive networks, increasing electrode resistance and degrading signal-to-noise ratios in electrochemical measurements. Conversely, excessive thermal processing may damage previously deposited biological layers or cause delamination of multilayer sensor architectures. These effects are particularly critical in wearable biosensors, where device performance depends on stable electrode–electrolyte interfaces and reliable signal transduction under continuous mechanical deformation. As a result, the development of low-temperature sintering strategies has become a central challenge in printed bioelectronics. Techniques such as photonic sintering, laser-assisted curing, chemical sintering, and plasma processing enable localized or transient heating that selectively densifies conductive inks while preserving surrounding biological components. These approaches provide a pathway for integrating high-performance conductive electrodes with temperature-sensitive functional materials, thereby enabling scalable fabrication of wearable and implantable biosensing platforms.

### 3.8. Practical Challenges in Sintering-Compatible Integration of Bio-Functional Layers

A critical challenge in printed biosensor fabrication is the integration of bio-functional layers with conductive electrodes that require post-print sintering. Biological recognition elements such as enzymes, antibodies, aptamers, and ion-selective membranes are typically sensitive to temperature, pH changes, and chemical environments [16,20,52,209,210]. As a result, the sequence of device fabrication becomes crucial. In most printed biosensor architectures, conductive electrodes are first printed and sintered before deposition of biological components to avoid thermal degradation [179,193].

Even with this approach, several challenges remain. Differences in thermal expansion coefficients between metallic electrodes and polymer substrates can lead to delamination or cracking during sintering. Additionally, incomplete removal of organic ligands or stabilizers from inks can interfere with subsequent bio-functionalization processes by inhibiting biomolecule attachment or altering surface chemistry [31,92]. Surface roughness and porosity introduced during sintering can also influence the stability of immobilized biomolecules, affecting sensor sensitivity and lifetime [211].

Maintaining adhesion between multiple functional layers such as electrodes, selective membranes, hydrogel layers, and encapsulation materials is, therefore, a critical design consideration [78,80]. Low-temperature sintering strategies that minimize thermal stress and chemical degradation are particularly important for ensuring stable integration of biological components in wearable and implantable biosensors [16,212,213].

## 4. Applications

In printed biosensors, device performance is strongly influenced by the interplay between ink chemistry, sintering conditions, and electrode microstructure. Conductive inks typically consist of metallic nanoparticles, metal–organic precursors, or hybrid conductive polymers that must be converted into electrically conductive networks through a sintering step. The chemical composition of the ink, including particle size, stabilizing ligands, and solvent systems, determines the temperature and energy required for particle coalescence. These parameters in turn influence the resulting microstructure of the printed electrode, including porosity, grain connectivity, and surface roughness.

These microstructural characteristics directly impact biosensor performance metrics. Highly conductive and uniform films reduce electrical resistance and improve signal stability, which is particularly important for electrophysiological sensors such as ECG (electrocardiography) or EEG (electroencephalogram) electrodes. In contrast, partially porous electrode structures can enhance electrochemical surface area, improving sensitivity in amperometry and enzymatic biosensors. Consequently, the combination of ink formulation and sintering strategy plays a central role in determining sensitivity, response time, and long-term stability of printed biosensors.

In printed biosensors, device architecture and sensing mechanisms play a crucial role in determining the requirements for ink formulation and sintering processes. Most electrochemical biosensors employ a three-electrode configuration consisting of a working electrode, reference electrode, and counter electrode. The working electrode is typically functionalized with catalytic or recognition materials that interact selectively with the target analyte, while the reference electrode maintains a stable potential and the counter electrode completes the electrochemical circuit. In wearable formats, these electrodes are often integrated into flexible multilayer structures that include microfluidic channels, selective membranes, and encapsulation layers. The performance of printed biosensors is strongly influenced by the interplay between ink chemistry, sintering conditions, and device architecture. Ink formulation determines nanoparticle size, ligand composition, and precursor decomposition pathways, which in turn govern the microstructure and electrical properties of the sintered electrode [53,54]. For example, smaller nanoparticles enable lower sintering temperatures due to melting point depression but require efficient ligand removal to achieve high conductivity [9]. Similarly, reactive precursor inks can produce dense metallic films at reduced temperatures but may introduce residual impurities that affect electrochemical stability. Consequently, the selection of appropriate ink chemistry and sintering strategy is a critical design parameter that directly impacts biosensor sensitivity, response time, and operational stability.

In this chapter, we focus our discussion on biochemical, biophysical, and electrophysiological sensing. These advancements are particularly critical when utilizing heat-sensitive substrates such as textiles and thin-film polymers, which have become the standard for next-generation wearables, on-skin “electronic tattoos,” and implantable bioelectronics. The transition from the lab to real-world applications imposes rigorous requirements on material performance. Specifically, properties such as conductivity, capacitance, and dielectric performance must remain stable under various operational conditions, including repetitive mechanical deformation (stretching and bending), varying ambient humidity, and prolonged exposure to corrosive body fluids (sweat). The discussion is categorized into three primary modalities: (i) biochemical sensing for the real-time analysis of molecular biomarkers, analyte-specific chemical transduction; (ii) biophysical sensing for physical-state transduction; and (iii) electrophysiological monitoring for capturing the body’s internal electrical signals.

Despite sharing similar fabrication strategies, biochemical, biophysical, and electrophysiological biosensors place different demands on conductive electrodes and, therefore, require distinct optimization of ink formulation and sintering processes. Biochemical sensors, such as glucose or lactate biosensors, rely on efficient electron transfer between enzymes and electrodes. In these systems, moderate surface roughness and controlled porosity produced by low-temperature sintering can enhance catalytic activity and improve sensitivity.

Biophysical sensors, including pressure or strain sensors used in wearable health monitoring, prioritize mechanical flexibility and stable electrical resistance under repeated deformation. For these devices, conductive networks produced through mild thermal or photonic sintering are preferred because they maintain conductivity while preserving the elasticity of the substrate.

Electrophysiological sensors, such as EEG, ECG, or EMG electrodes, require highly conductive and low-noise electrode interfaces to accurately capture weak bioelectrical signals. In these cases, dense conductive films produced by photonic or laser sintering are often advantageous because they minimize contact resistance and improve signal fidelity. These differences illustrate how the optimal sintering strategy depends on the sensing mechanism and device architecture used in a particular biosensor application.

### 4.1. Biochemical Sensors

Biochemical sensors can be wearable and printable; they directly target specific metabolites, ions, or volatile biomarkers through chemical recognition and transduction mechanisms. In these devices, signal generation arises from enzymatic catalysis, electrocatalytic oxidation/reduction, potentiometric ion exchange, or chem-resistive adsorption processes. The integration of Ag and Cu nanoparticle inks plays a dual role: providing low-resistance conductive pathways and reference electrodes (Ag/AgCl), while also enabling catalytic working electrodes in enzyme-free configurations (e.g., CuO-based systems). The following subsections discuss key biochemical sensing modalities, including metabolite detection (glucose, lactate, and cholesterol), pH sensing, alcohol and acetone monitoring, and gas-based biomarkers, with emphasis on device architecture, performance metrics, and materials compatibility, which are summarized in Table 4.

(1) Biological Fluid-Based Sensors

Biological fluid–based sensing platforms represent the systems-level framework in which printed conductive materials, reference electrodes, catalytic layers, and functional membranes must operate cohesively. Unlike single-analyte electrochemical cells, these platforms must address fluid acquisition, transport control, biofouling resistance, evaporation mitigation, and reference stability in complex and dynamic matrices such as sweat, saliva, wound exudate, and interstitial fluid (ISF). As reviewed by Gao et al., wearable electrochemical systems have evolved toward fully integrated patches that combine printed metallic electrodes, microfluidic routing, and multiplexed sensing modalities to manage these constraints while maintaining mechanical flexibility and signal fidelity [214]. An example of integrated device engineering is the fully autonomous sweat platform reported by Emaminejad et al., which combined screen-printed Ag/AgCl reference electrodes, patterned Au working electrodes, and microfluidic sweat routing within a soft wearable architecture [215]. The device incorporated iontophoresis-based sweat induction and on-board electronics for real-time readout. Materials-wise, Ag inks were critical for low-resistance interconnects and stable pseudo-reference functionality, while microfluidic reservoirs mitigated dilution artifacts. Device-level validation included correlation to clinical assays (chloride and glucose), demonstrating that integration of fluidics and electrochemistry substantially improves analytical reliability compared to passive diffusion-based designs [215]. Temporary-transfer tattoo sensors introduced by Jia et al. demonstrated the feasibility of ultra-thin, conformal Ag-based printed electrodes laminated directly onto the epidermis for lactate detection in perspiration [216]. The device employed screen-printed Ag/AgCl reference electrodes and enzyme-modified working electrodes fabricated at low temperature to preserve bioactivity. Compared to rigid sensor formats, the tattoo configuration minimized motion artifacts and improved electrode–skin coupling. However, long-term stability was limited by dehydration and enzyme degradation, illustrating the tradeoff between mechanical compliance and operational longevity [216]. Reference electrode engineering remains a central limitation in biological fluid platforms. Sophocleous and Atkinson critically evaluated screen-printed Ag/AgCl reference electrodes, showing that chloride depletion, junction instability, and limited electrolyte reservoirs can induce potential drift under continuous operation [217]. For wearable sweat systems, where chloride concentration fluctuates significantly, pseudo-reference designs must incorporate either salt reservoirs or encapsulated hydrogel layers to stabilize activity. Comparative studies indicate that, while printed Ag/AgCl references offer manufacturing simplicity, their drift (often >2–5 mV h^−1^ under flow) can dominate total measurement uncertainty unless compensated [217]. More recent microfluidic architectures, such as the bioinspired sweat sampling platform by Shin et al., integrate continuous-flow microchannels, capillary burst valves, and metabolite-specific electrochemical cells to enable multiday sampling with reduced evaporation artifacts [218]. Fabrication involved multilayer soft lithography combined with printed metallic electrodes. Device-level testing demonstrated improved temporal resolution and reduced concentration artifacts compared to static patch designs. Such systems highlight the importance of coupling sampling physics with electrochemical design, rather than treating electrodes as standalone components. From a materials perspective, Ag nanoparticle inks remain the dominant choice for conductive traces and reference electrodes due to their high conductivity and compatibility with low-temperature curing (<150 °C or photonic sintering). Cu-based nanoparticle inks offer cost advantages and catalytic utility in enzyme-free assays but are less stable in chloride-rich sweat unless passivated or encapsulated. In multiplexed platforms, the sequence of fabrication is critical: metallic traces are typically printed and sintered first, followed by deposition of enzyme layers, ion-selective membranes, or hydrogel encapsulants to preserve functional integrity. Comparatively, platform performance is now evaluated not only by sensitivity and limit of detection but also by system-level metrics: drift over 24–72 h, response under bending cycles (>1000 cycles), calibration transfer from in vitro to on-body conditions, and error propagation due to sweat-rate variability. Devices incorporating active fluid control and stabilized references consistently outperform passive single-layer patches in terms of reproducibility and correlation to gold-standard assays. However, standardization remains limited; artificial sweat formulations, flow rates, and validation protocols vary widely across studies, complicating cross-platform comparison.

(2) Metabolite Sensor (Glucose and Lactate Sensors)

Glucose and lactate represent the two most extensively studied metabolites in wearable electrochemical biosensing, collectively establishing the materials and architectural principles that underpin printed biofluid diagnostics. Foundational electrochemical work by Wang and Heller defined enzymatic oxidase-based sensing as the gold standard for specificity and clinical relevance [27,51]. In wearable contexts, however, translation from blood to sweat or tears introduces additional constraints: metabolite concentrations are lower (particularly for glucose), secretion kinetics are variable, and sampling artifacts can dominate signal interpretation. As such, wearable metabolite sensors are fundamentally systems-level devices in which materials processing, microfluidics, and reference stability are as critical as catalytic chemistry. Enzymatic architectures that use glucose oxidase (GOx) for glucose and lactate oxidase (LOx) for lactate remain the dominant strategy because they offer molecular specificity and compatibility with low-potential amperometric detection. Screen- or inkjet-printed Ag nanoparticle inks enable low-resistance conductive traces and Ag/AgCl pseudo-reference electrodes, which are typically fabricated first and sintered at low temperature prior to bio-functionalization. The temporary-transfer lactate tattoo by Jia et al. demonstrated conformal epidermal integration using printed Ag electrodes [216], while the autonomous sweat platform of Emaminejad et al. integrated printed electrodes with microfluidic routing for glucose analysis [215]. These device-level examples illustrate that mechanical compliance, reference stabilization, and fluid control determine analytical robustness as much as enzymatic sensitivity. Non-enzymatic approaches, particularly CuO-based glucose electrodes demonstrated via inkjet printing (Ahmad et al.), offer attractive scalability and eliminate enzyme shelf-life limitations [212]. Cu-based nanostructures can catalyze glucose oxidation over wide linear ranges, and similar catalytic concepts are being explored for lactate [219]. However, in chloride-rich sweat matrices, Cu surface evolution and higher operating potentials introduce baseline drift and increased susceptibility to interferents. Consequently, while non-enzymatic systems simplify materials stacks, they often require greater calibration control and protective membrane engineering to achieve wearable reliability.

From a comparative perspective, glucose sensing emphasizes sensitivity and drift control due to lower biofluid concentrations, whereas lactate sensing serves as a robustness benchmark because of higher sweat levels during exertion. Enzymatic systems generally outperform Cu-based alternatives in selectivity and low-potential operation, particularly under dynamic on-body conditions. Nevertheless, Cu-based strategies remain compelling for long-term storage stability and large-area manufacturing, provided that surface passivation and chloride stability are improved. Low-temperature fabrication is the central materials constraint unifying both metabolite classes. Conductive Ag traces must be sintered without exceeding enzyme tolerance, reinforcing a “print-sinter-functionalize” workflow. Photonic or chemical sintering methods are, therefore, preferred to preserve bioactivity while achieving adequate conductivity. The integration of microfluidics for sweat-rate normalization, temperature compensation, and time-resolved sampling further enhances metabolite interpretation, as concentration changes can reflect both systemic metabolism and local secretion dynamics. Ultimately, wearable metabolite sensing is not defined by catalytic sensitivity alone but by the coupled optimization of conductive inks, reference electrode engineering, enzyme stabilization, sampling physics, and mechanical design. Glucose and lactate platforms together demonstrate that reliable translation from bench electrochemistry to epidermal diagnostics requires materials science, device architecture, and physiological context to be engineered as an integrated whole.

(3) pH Sensor

pH sensing is foundational in wearable biofluid diagnostics because it serves both as a physiological biomarker and as a correction parameter for enzyme kinetics and redox-based metabolite detection. In sweat analysis, pH reflects skin barrier integrity, irritation state, and disease-associated changes, while in microfluidic and organ-on-chip systems, it provides a real-time indicator of metabolic activity. Unlike enzymatic metabolite sensors, potentiometric pH devices are fundamentally limited by reference electrode stability, making materials engineering central to performance. Screen-printed Ag/AgCl electrodes remain the dominant reference architecture due to scalability and fabrication simplicity. However, as highlighted by Sophocleous and Atkinson, thin-film Ag/AgCl references are susceptible to chloride depletion, junction instability, and drift effects that become pronounced in variable sweat environments [217]. Advances such as the compact, long-term-stable Ag/AgCl reference developed by Dawkins et al. demonstrate that microstructural control, encapsulation, and chloride management significantly improve stability [220]. These improvements are especially important when pH sensing is co-integrated with glucose or lactate channels, where reference drift propagates into apparent metabolite error. Inkjet-printed pH sensors have extended applicability into organ-on-chip platforms, where miniaturization and continuous perfusion stability are paramount. Boček et al. demonstrated flexible inkjet-printed pH electrodes compatible with microfluidic biomedical testing, emphasizing low-temperature processing and geometric precision [221]. In contrast, wearable colorimetric textile systems, such as those reported by Ha et al., eliminate the need for reference electrodes and power but sacrifice quantitative precision and multiplexing capability [222]. Electrochemical sensors typically approach Nernstian slopes (~59 mV/pH) with rapid response, whereas optical systems prioritize simplicity and mechanical robustness. From a fabrication perspective, compatibility with biological functional layers and adjacent sensor channels is critical. High-temperature sintering of conductive inks can damage adjacent membranes or enzyme layers; therefore, low-temperature curing and sequential processing are essential for reproducibility. Ultimately, in wearable pH sensing, reference electrode engineering and drift control, not the pH-sensitive layer itself, are the dominant determinants of long-term accuracy. For rigorous reporting, calibration slope, drift (mV h^−1^), hysteresis, and stability in defined artificial sweat matrices should be explicitly quantified.

(4) Cholesterol Sensor

Cholesterol sensing remains a high-value objective in biosensor development due to its central role in cardiovascular disease and metabolic disorders. Unlike hydrophilic metabolites such as glucose and lactate, cholesterol is hydrophobic and typically associated with lipoproteins, necessitating surfactant-assisted solubilization or membrane engineering to generate an electroactive signal. As reviewed by Narwal et al., enzymatic cholesterol oxidase (ChOx)-based systems dominate established electrochemical platforms, converting cholesterol into hydrogen peroxide for amperometric detection [223]. In printed formats, screen-printed electrodes, often Au, Pt, or carbon working electrodes combined with Ag/AgCl references and Ag interconnects, enable low-cost disposable assays. Device-level comparisons highlight the importance of electrode material and fabrication methods. Shen et al., demonstrated that screen-printed Au and Pt working electrodes exhibit different peroxide oxidation efficiencies, influencing sensitivity and response kinetics [224]. These results underscore that electrode composition, nano-structuring, and self-assembly strategies directly shape biosensor performance beyond enzymatic specificity alone. Non-enzymatic approaches aim to overcome enzyme instability and storage constraints. Cu_2_O-decorated TiO_2_ nanotubes by Khaliq et al. [225], and hybrid Ag–Cu_2_O nanostructures by Kumar et al. [226], illustrate how nanostructured oxides and conductive Ag incorporation enhance catalytic activity and charge transport. However, as highlighted by Sha et al., selectivity and fouling in protein-rich matrices remain major translational barriers for metal-based systems [227]. While these materials demonstrate promising sensitivity in buffered electrolytes, real-sample performance depends heavily on surface passivation and interference control. Comparatively, enzymatic sensors retain superior specificity and clinical validation, particularly for serum assays, whereas non-enzymatic Cu-based systems offer improved thermal robustness and simplified storage but require advanced nano-structuring and calibration strategies. For wearable or saliva-based platforms, the central challenge is integrating cholesterol solubilization and pre-treatment without compromising comfort, flexibility, or long-term stability. Low-temperature fabrication constraints are parallel to those in other bio-functional devices: conductive Ag traces must be sintered before enzyme deposition, and solvent exposure must be minimized to preserve membrane integrity. Ultimately, successful cholesterol sensing hinges not only on catalytic performance but on matrix-aware engineering, balancing material robustness, selective interfaces, and device architecture to enable reliable operation in complex biological environments.

(5) Acetone (Diabetes-Related) Sensors

Acetone is a clinically relevant volatile organic compound (VOC) associated with fat metabolism and diabetic ketosis, making breath acetone sensing an attractive route toward noninvasive metabolic monitoring. Unlike liquid-phase electrochemical metabolite sensors, wearable acetone detection typically relies on chemi-resistive metal oxide semiconductors, most commonly ZnO or CuO, patterned onto flexible substrates. In these architectures, printed Ag or Cu nanoparticle inks serve as electrodes and often as integrated microheaters, enabling scalable fabrication via screen or inkjet printing. CuO-based sensing layers are particularly promising because they can be formulated from nanoparticle inks compatible with flexible processing. Aydin et al. demonstrated flexible CuO-based conductometric sensors fabricated on polymer substrates, highlighting the suitability of solution-processed oxides for wearable integration [228]. Similarly, flexible Kapton-based platforms integrating Cu-based conductive traces and gas-sensitive films illustrate how polymer-compatible printing can enable lightweight acetone sensors [229]. However, oxide performance typically depends on thermal activation, and polymer substrates constrain post-deposition annealing temperatures. Selectivity remains the central challenge. Chen et al. demonstrated that temperature modulation of hierarchical ZnO sensors enhances acetone discrimination at breath-level concentrations [230]. Such approaches rely on printed microheaters, often Ag-based due to low resistivity, to generate dynamic response signatures. Yet, heated oxide layers introduce significant power demands, and as emphasized in wearable respiratory sensor reviews by Yin et al. [231], energy efficiency is critical for practical deployment. Strategies such as duty cycling, RFID powering, and nanostructured oxides that operate at reduced temperatures are, therefore, central to translation. Performance must be evaluated under humidified synthetic breath rather than ideal dry gas streams, as humidity strongly influences metal oxide response. Clinically relevant breath acetone levels lie in the sub-ppm to low-ppm range, and robust cross-sensitivity testing against ethanol and other VOCs is essential. Baseline recovery, long-term drift, and repeatability under cyclic exposure ultimately determine wearable feasibility. Low-temperature processing presents both a constraint and an opportunity: photonic or chemical sintering enables flexible Ag electrodes and microheaters, while low-temperature-matured nanostructured oxides offer pathways to reduce energy consumption. Ultimately, successful printed acetone sensors will require integrated optimization of catalytic nanomaterials, power management, humidity mitigation, and validation against gold-standard methods such as GC–MS, bridging the gap between laboratory demonstrations and clinically meaningful wearable diagnostics.

(6) Alcohol Detection Sensors

Wearable alcohol monitoring has emerged as a promising noninvasive approach for tracking consumption patterns, supporting behavioral interventions, and enabling clinical management of alcohol use disorders. Sweat-based sensing is particularly attractive because it allows continuous monitoring without breath sampling. Two primary strategies dominate: direct ethanol detection, either via enzymatic alcohol oxidase (AOx) systems or catalytic electro oxidation, and indirect detection of ethyl glucuronide (EtG), a stable ethanol metabolite that extends the detection window. Screen-printed electrochemical platforms remain the dominant fabrication strategy for wearable alcohol sensors due to their scalability and compatibility with Ag nanoparticle inks. Ag-based conductive traces and Ag/AgCl reference electrodes are typically printed and sintered first, forming the electrical backbone of the device. Campbell et al. reviewed wearable electrochemical alcohol biosensors and emphasized that Ag/AgCl pseudo-references provide low-cost integration but require careful stabilization in sweat matrices where ionic strength fluctuates [232]. In enzyme-based ethanol sensors, the working electrode is subsequently modified with alcohol oxidase or catalytic films embedded in hydrogels, enabling peroxide-mediated detection. Direct electrochemical monitoring of ethanol in sweat has been demonstrated using printed electrode stacks optimized for diffusion control and stability. Biscay et al. reported electrochemical alcohol detection in sweat, highlighting the importance of membrane engineering to regulate analyte flux and reduce interference from uric acid and other electroactive species [233]. Device-level evaluation showed that signal drift, rather than peak sensitivity, determines long-term monitoring viability. Evaporation-induced concentration artifacts and protein fouling were identified as key failure modes, underscoring the need for encapsulation strategies that maintain hydration while permitting analyte ingress. EtG detection offers improved specificity because EtG persists longer than ethanol and is directly linked to alcohol consumption events. Selvam et al. demonstrated a wearable biochemical sensor capable of EtG detection in human sweat using affinity-based recognition integrated into a wearable platform [234]. The device architecture incorporated printed conductive layers and bio-functional sensing regions capable of quantifying EtG over time, correlating sensor output with controlled consumption events. However, incorporation of antibodies or affinity reagents increases fabrication complexity and introduces additional stability considerations. Recent advances in selectivity engineering are exemplified by Ma et al., who reported highly selective wearable alcohol homologue sensors capable of distinguishing ethanol from related alcohols [235]. These systems leveraged tailored catalytic surfaces and electrochemical protocols to enhance discrimination, demonstrating that materials design at the electrode interface is central to specificity. Such strategies are particularly relevant in sweat, where interfering compounds and environmental contaminants can confound measurement. Comparatively, direct ethanol detection offers real-time responsiveness but is sensitive to evaporation effects and transient fluctuations, whereas EtG-based sensing provides higher specificity and extended detection windows at the cost of increased fabrication complexity. For wearable translation, the decisive metrics extend beyond the limit of detection to include dynamic range within sweat-relevant concentrations, selectivity against common sweat constituents, multi-day drift stability, and correlation with reference measures such as blood alcohol concentration (BAC) or laboratory EtG assays. Calibration transfer between users remains a significant challenge due to variability in sweat rate and skin physiology. Low-temperature processing is critical because alcohol sensors frequently incorporate enzymes, antibodies, or hydrogel matrices that are sensitive to thermal and chemical stress. Conductive Ag traces should be fully sintered prior to bio-functionalization, and post-processing residues from plasma treatments or chemical sintering must be evaluated for compatibility with the biolayer. Ultimately, successful wearable alcohol sensors require integrated optimization of conductive ink stability, reference electrode engineering, diffusion control layers, and biorecognition element preservation to achieve reliable, real-world monitoring performance.

**Table 4:** Representative biochemical biosensors fabricated using low-temperature sintering inks and their key performance metrics.

Ink Material, Electrodes, and their role	Printing and Processing Conditions	Substrate	Sensing Mechanism/Transduction	Performance Metrics	Target Analyte -- Sensor Type
Ag/AgCl reference electrodes, gold working electrodes, and carbon counter electrodes integrated with enzyme-functionalized sensing layers (e.g., glucose oxidase)	Screen printing/microfabrication, low temperature curing <100 °C	PET/flexible polymer	Potentiometric ion-selective electrode (ISE) for chloride detection, Enzymatic amperometric detection	Real-time monitoring of sweat biomarkers during sedentary conditions; demonstrated quantitative correlation between sweat glucose and blood glucose trends	Multi-analyte (sweat/saliva) -- Biological fluid-based platforms [215]
Carbon working electrode ink + Ag/AgCl reference ink	Screen printing; curing 60–90 °C	Temporary tattoo/PET	Enzymatic amperometric detection using lactate oxidase	Linear response 0–20 mM lactate	Lactate (sweat) -- Lactate biosensor [216]
Conductive Ag ink and CuO catalytic WE	Inkjet; screen printing, CuO annealing: 150–300 °C	Flexible polymer	Electrocatalytic oxidation on CuO surface	Linear-detecting range of 0.05–18.45 mM and the detection limit of ∼0.5 μM (*S*/*N* = 3) sensitivity 2762.5 μAm M^−1^ cm^−2^	Glucose -- Glucose (CuO non-enzymatic) [212]
Ag/AgCl reference and CuO pH film (optional)	Screen printing; inkjet, 80–150 °C	PET	Potentiometric measurement based on Nernst equation	Near-Nernstian slope (~59 mV/pH); reference stability critical	pH (H^+^) -- pH (electrochemical) [222]
Ag ink for conductivity enhancement and Cu_2_O	Wet chemical bath deposition (CBD) technique, screen printing, and hybrid nanostructure deposition	200–400 °C (rigid); <200 °C (flexible variants)	Enzymatic electrochemical oxidation	Enzyme-free; nanostructure-dependent sensitivity; sensitivity (12,140.06 μAmM^−1^ cm^−2^) low detection limit (0.057 mM) and fast response	Cholesterol -- Cholesterol (enzymatic) [226]
Ag ink for interconnect	Screen printing, 100–150 °C; biolayer: RT	Temporary tattoo	Enzymatic amperometric detection	Direct vs. metabolite detection; multi-day monitoring; drift-sensitive	Ethanol/EtG -- Alcohol (enzymatic/EtG) [234]
Ag electrodes and CuO sensing film	Inkjet; screen printing 150–350 °C (oxide); photonic curing preferred	Flexible polymer	Chemi-resistive gas sensing via surface adsorption	Sub-ppm detection; humidity sensitivity; temperature modulation	Acetone -- Acetone (metal oxide) [230]

Finally, performance benchmarks from electrochemical genosensors further define the clinical targets that low-temperature, printed biosensor platforms must meet for real-world deployment. A paper-based electrochemical genosensor was developed for SARS-CoV-2 RNA detection with sub-5 min response time, a limit of detection of 6.9 copies µL^−1^, and a broad linear range from ~10^3^ to >10^7^ copies µL^−1^, achieving near-100% accuracy, sensitivity, and specificity when validated against 48 clinical samples [236]. Although not fabricated using conductive inks, this work defines the speed, sensitivity, and robustness benchmarks that emerging printed and ink-based biosensors must satisfy real-world deployment.

### 4.2. Biophysical Sensing

Biophysical sensors measure changes in physical properties such as temperature, strain, pressure, impedance, or hydration rather than specific chemical reactions. These devices are essential for contextualizing biochemical signals and for directly monitoring physiological or mechanical states of the body. In printed wearable platforms, Ag and Cu nanoparticle inks primarily function as conductive traces, resistive elements, or interdigitated electrode structures. Performance is governed by mechanical durability, signal stability under deformation, and environmental robustness. The following section examines hydration, temperature, motion, and pressure/strain sensors, highlighting device-level engineering considerations and integration strategies within multimodal wearable systems. This section, therefore, highlights recent advances in the fabrication of biophysical sensing electrodes using additive manufacturing and printing techniques combined with low-temperature and chemically assisted sintering, with representative examples summarized in Table 5.

(1) Hydration and Dehydration Sensors

Wearable hydration monitoring translates physical electrical properties—such as skin impedance, dielectric constant, or conductance—into estimates of fluid status and sweat loss. Unlike biochemical sensors, hydration devices rely on indirect proxies and are, therefore, highly sensitive to electrode contact pressure, ambient humidity, and calibration assumptions. Recent reviews highlight a shift from simple conductometric patches toward impedance-based systems and microfluidic integration to improve reliability and physiological interpretation [237]. Printed interdigitated electrodes (IDEs) fabricated from Ag nanoparticle inks are central to impedance-based hydration sensors due to their fine patterning capability and low-resistance performance at flexible-compatible curing temperatures (80–150 °C). Es Sebar et al. demonstrated wearable EIS-based hydration monitoring in elderly patients using IDE architectures, showing that frequency-dependent impedance can reflect hydration-related changes [238]. However, these measurements primarily probe local skin barrier properties, necessitating careful distinction between superficial moisture and systemic hydration. Microfluidic sweat-rate sensing offers a complementary strategy by directly quantifying fluid loss. Ursem et al. developed wearable capillary-driven microfluidic devices capable of tracking sweat flow rates [239], reducing confounding from ionic strength variations that can distort purely electrical measurements. Integration of impedance sensing with sweat-rate microfluidics represents a promising dual-parameter approach for more accurate hydration assessment. From a materials perspective, Ag inks remain preferred for IDE fabrication due to stability in humid environments, while Cu-based traces require encapsulation to mitigate oxidation. The dominant engineering challenge often lies not in electrode conductivity but in encapsulation and adhesive design, which must maintain stable contact without trapping moisture or introducing hysteresis. For rigorous evaluation, hydration sensors should report equivalent circuit models, calibration protocols across controlled dehydration states, and validation against reference metrics such as body mass change or plasma osmolality. Ultimately, successful wearable hydration monitoring depends on integrating stable printed electrodes, environmental compensation, and microfluidic normalization to bridge local electrical measurements with systemic fluid balance.

(2) Temperature sensors

Skin temperature is a fundamental physiological parameter in wearable systems, serving both as an independent biomarker (fever detection, thermal stress, wound monitoring) and as a correction factor for enzyme-based electrochemical assays whose kinetics are temperature dependent. In multimodal wearable platforms, temperature sensing is increasingly integrated alongside glucose, lactate, and pH channels to improve analytical accuracy rather than functioning as a standalone readout. The feasibility of ultra-thin, conformal temperature monitoring was established in early epidermal electronics platforms, where serpentine metallic traces maintained conductivity under mechanical deformation while providing accurate thermal measurement [20]. This work defined the design paradigm for printed temperature sensors: low thermal mass, intimate skin contact, and mechanically compliant geometries. In printed implementations, Ag nanoparticle inks are widely used to fabricate resistive meanders or interdigitated electrodes due to their high conductivity and compatibility with low-temperature curing (≈80–150 °C or photonic sintering). Recently, Al-Qahtani et al. designed a wearable combined Ag electrodes with carbon-based sensing films or composite resistive layers to enhance sensitivity and stability [240]. Reviews of printed biomedical temperature sensors emphasize that while Cu-based traces offer cost advantages, oxidation in humid and sweat-rich environments can introduce drift that mimics thermal changes [241]. Consequently, Ag remains the preferred material for stable long-term monitoring in wearable contexts. From a translational perspective, performance evaluation should prioritize calibrated accuracy (±0.1–0.3 °C), response time, hysteresis during thermal cycling, and robustness under bending and sweat exposure. Low-temperature processing is critical, enabling direct printing onto textiles and polymer substrates and allowing monolithic integration with enzymatic biosensors without compromising bioactivity. Ultimately, temperature sensors in wearable systems are most impactful when explicitly incorporated into compensation algorithms for biochemical assays, demonstrating how integrated thermal readouts reduce analytical error and enhance clinical reliability.

(3) Motion and activity sensors

Motion sensing in printed wearable systems is primarily realized through skin-interfaced strain gauges fabricated from conductive metal inks, enabling applications in rehabilitation, gait analysis, sports performance, and human–machine interfaces. The design principles for conformal motion sensing were established in early epidermal electronics, where serpentine metallic traces maintained electrical continuity under bending and stretching [20]. This geometry-driven strategy remains central to printed strain sensors today. Ag nanoparticle inks are widely used for strain gauge fabrication due to their high conductivity and compatibility with low-temperature sintering (80–150 °C), allowing direct printing onto flexible polymers, textiles, and adhesive substrates. Reviews of printed strain sensors emphasize that material selection, pattern geometry, and fabrication method (screen printing and aerosol jet) collectively determine gauge factors, strain window, and fatigue life [242]. Recent advances in nano-silver conductive ink strain sensors demonstrate how microstructural engineering enhances performance. Chi et al. reported flexible strain sensors fabricated from printed nano-silver inks exhibiting tunable gauge factors and improved mechanical durability [30]. By optimizing ink formulation and film morphology, they achieved stable resistance changes across repeated deformation cycles. Such nano-engineered films can balance sensitivity and fatigue life, though higher gauge factors often correlate with increased hysteresis due to microcrack formation or percolation network rearrangement. Comparatively, serpentine metal traces provide moderate sensitivity with excellent cyclic stability, whereas microcracked or composite networks achieve higher gauge factors at the cost of long-term repeatability. Key performance metrics should include gauge factor, hysteresis, response time, drift, and fatigue life over ≥10^3^–10^5^ cycles. Importantly, manuscripts should distinguish intrinsic sensor physics (signal quality and linearity) from downstream machine learning classification performance [242]. In multimodal wearable platforms, motion signals provide essential contextual information, improving artifact rejection and enabling correlation between activity intensity and biochemical markers such as lactate. Ultimately, motion sensing in wearable biosystems is governed as much by geometric and packaging design as by conductive ink formulation.

(4) Pressure and strain sensors

Pressure and strain sensors in wearable systems extend motion sensing into the domain of contact force measurement, enabling applications such as plantar pressure mapping, grip-force quantification, posture monitoring, and distributed tactile sensing in electronic skin. While strain gauges detect tensile deformation, pressure sensors typically transduce compressive force through piezoresistive composites, capacitive microstructured dielectrics, or patterned metal films designed for small-deformation regimes. In printed wearable platforms, these devices are often fabricated via screen printing or related additive techniques that enable scalable patterning on flexible substrates. The foundational feasibility of mechanically compliant skin-conformal electronics was established in early epidermal electronics work [20], where serpentine metallic interconnects accommodated deformation without fracture. This geometric strategy informs modern printed pressure/strain sensors, in which Ag nanoparticle inks are widely used to create low-resistance electrodes and high-density interconnects compatible with elastomeric or textile substrates. Low-temperature curing (80–150 °C or photonic sintering) enables direct integration with soft materials while preserving mechanical flexibility. Printing-based fabrication approaches have advanced significantly, as reviewed by Qi et al., who summarized flexible strain sensors fabricated through screen printing, inkjet printing, and related methods [243]. Printed metal traces can serve as resistive strain gauges, while conductive filler–elastomer composites (e.g., carbon or metal particles in PDMS) provide piezoresistive pressure transduction. Composite approaches often offer higher sensitivity but are susceptible to viscoelastic creep and baseline drift under repeated loading. In contrast, metal-film strain gauges provide stable resistance changes in small-strain regimes but may have limited stretchability unless they are patterned into serpentine geometries. Device-level translation highlights the importance of skin compatibility and packaging. Rauf et al. demonstrated fully screen-printed, gentle-to-skin electrodes integrated with miniature PCBs for wearable electrophysiological monitoring [244]. Although focused on ECG, this work illustrates how printed electrode arrays can maintain stable contact under mechanical loading, a principle equally relevant to pressure-sensing arrays integrated into healthcare textiles. For applications such as ulcer prevention or posture monitoring, biocompatibility, breathability, and washability often determine real-world adoption more than peak sensitivity. Comparative performance analysis should include sensitivity (ΔR/ΔP or ΔC/ΔP), minimum detectable pressure, response and recovery times, hysteresis during cyclic loading, and cycling stability over ≥10^3^–10^5^ load cycles. Piezoresistive composites frequently exhibit higher sensitivity but greater hysteresis and drift due to elastomer creep, whereas printed metal strain gauges offer lower hysteresis but a narrower dynamic range. Cu-based conductive inks may reduce material cost but require encapsulation to mitigate oxidation and adhesion loss on soft substrates; many fabrication strategies, therefore, favor Ag inks for long-term stability in humid, sweat-exposed conditions. Low-temperature sintering is enabling for integrating metallic electrodes with elastomers and textiles, and photonic curing minimizes thermal damage to soft substrates. However, residual solvents or sintering additives can migrate into elastomer matrices and alter mechanical response, affecting calibration stability. Careful materials compatibility testing is, therefore, essential.

**Table 5:** Printed biophysical sensors fabricated using low-temperature conductive inks with their processing temperatures and sensing mechanisms.

Ink Material, Electrodes, and Their Role	Printing and Processing Conditions	Substrate	Measured type	Sensing Mechanism/Transduction	Performance Metrics	Biosensor Type
Ag Interdigitated electrodes	Screen printing 80–150 °C	PET	Skin conductivity by Skin hydration	Impedance-based hydration tracking; sweat-rate dependent	Real-time hydration monitoring	Hydration/Impedance [238]
Ag Resistive serpentine heater and Cu resistive traces (oxidation risk)	Screen printing; inkjet 100–150 °C	Elastomer	Skin temperature	Thermoresistive sensing; fast response; low power	Sensitivity ~0.01 °C	Temperature [20,240]
Ag Stretchable conductive traces	Screen printing; aerosol jet 80–150 °C	Textile	Mechanical deformation. Motion/bending	High gauge factor; fatigue durability; flexible substrates	High durability (>1000 cycles)	Motion/Strain [30,242]
Ag electrode arrays and Cu Conductive interconnects	Screen printing; aerosol jet 80–150 °C	Elastomer	Mechanical force	Piezoresistive/capacitive modes; wearable integration	Gauge factor 10–20	Pressure [243,244]

### 4.3. Electrophysiological Sensors

Electrophysiological (EP) signals are low-level changes in electrical potential generated by the activity of human organs and tissues and have long been of interest in healthcare and human–machine interface applications [245,246,247]. These signals include electrocardiogram (ECG) [248], electrooculogram (EOG) [249], Photoplethysmography (PPG) [250], electromyogram (EMG) [211], and electroencephalogram (EEG) [251]. Recent advances in printed and soft electronics have enabled a new generation of wearable EP that emphasize conformal skin contact, scalable manufacturing, and wireless integration.

A central technological enabler of these platforms is the development of low-temperature sintering conductive inks, which allow highly conductive electrodes and interconnects to be formed on thermally sensitive, skin-compatible substrates such as polyethylene terephthalate (PET), thermoplastic polyurethane (TPU), textiles, and elastomers without degrading polymers, adhesives, or bio-functional layers. By promoting interparticle fusion and percolation at mild temperatures or through chemically assisted pathways, these inks combine high electrical conductivity with mechanical compliance across diverse printing modalities. In wearable biosensors, they primarily function as low-impedance electrodes for EP signal acquisition (ECG, EMG, and EEG) and as stable electrochemical interfaces for metabolite and electrolyte sensing, where robust adhesion and resistance stability under repeated deformation are essential for long-term signal fidelity.

EP electrodes can be broadly classified as invasive or non-invasive, with this review focusing on non-invasive implementations. Among available material systems, Ag-based inks, including Ag nanoparticles, Ag nanowires, and stretchable Ag composites, are most widely adopted due to their low resistivity, printability, and compatibility with low-temperature sintering strategies. This section, therefore, highlights recent advances in the fabrication of physiological sensing electrodes using additive manufacturing and printing techniques combined with low-temperature and chemically assisted sintering, with representative examples summarized in Table 6 for applications in ECG, EEG, EMG, and EOG sensing.

(1) Electrocardiography (ECG)

**Figure 6:**
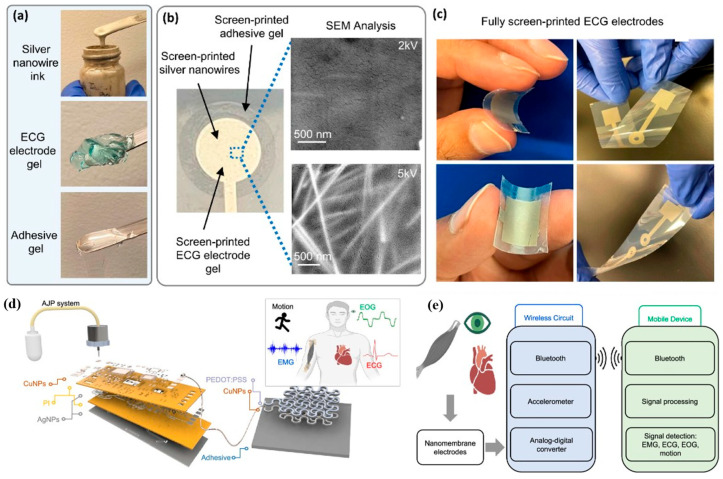
Electrophysiological sensor (ECG) application using low-temperature sintering inks; fabrication and characterization of ECG electrodes. (**a**) Ag NW Inks used for screen-printing of electrodes. (**b**) Electrode comprising conductive Ag NWs, ECG electrode gel, and adhesive gel screen-printed on a plastic substrate. (**c**) Flexible, fully screen-printed ECG electrodes. Reproduced with permission [244]. (**d**) AJP-based fabrication of integrated electronics using PI, AgNPs, CuNPs, and PEDOT:PSS for a multilayered circuit and an electrode and target physiological signals, measured by the fabricated wearable electronics, including EMG, ECG, EOG, and motion; (**e**) flowchart showing the overall processes of signal detection, starting from electrode mounting on targeted skin to wireless data transfer with a circuit and data processing on a mobile device. Reproduced with permission [254].

Electrocardiography (ECG) monitors the bioelectric activity of the heart by detecting depolarization and repolarization events that define clinically relevant features, such as the P wave, PR interval, QRS complex, and T wave [260]. Low-temperature sintering conductive inks enable the fabrication of high-performance ECG electrodes directly on flexible, skin-compatible substrates without thermal degradation. For example, hybrid chemical–electrophysiological patches have been realized by printing Ag/AgCl, Prussian blue, and insulating layers onto transparent polyester substrates, followed by mild thermal curing below 100 °C, preserving polymer integrity while achieving low-impedance, conformal ECG electrodes integrated with electrochemical sensing [252]. Similarly, fully printed ECG systems based on Ag nanowire and hybrid Ag nanowire/graphene oxide inks employ low-temperature post-treatment to form continuous, highly conductive networks on PET and polymer films, delivering signal quality comparable to commercial wet Ag/AgCl electrodes while supporting scalable manufacturing and wireless operation (Figure 6a–c) [244]. By combining AgNWs with graphene oxide (GO), as well as the screen-printing technology, the fabricated AgNWs/GO hybrid transparent conductive electrodes on PET substrate showed excellent mechanical flexibility [113]. Together, these studies highlight how low-temperature sintering and mild curing strategies are central to integrating high-fidelity ECG sensing with flexible, printable platforms for wearable and telemedicine applications.

(2) Electromyography (EMG)

Electromyography (EMG) measures the bioelectric activity of muscles during contraction and is widely used for human–machine interfaces, prosthetics, and wearable robotics. Low-temperature and chemically assisted sintering strategies have been utilized in producing highly conductive, mechanically compliant electrodes to be formed on soft, skin-compatible substrates without thermal damage. For example, printable elastic conductors based on Ag flakes dispersed in fluorinated elastomers exploit mild post-treatment and surfactant-directed network formation to achieve continuous, stretchable percolation pathways that retain high conductivity even beyond 200% strain, supporting stable textile-based EMG electrodes [112]. In inkjet-printed sEMG matrices, low-temperature nanoparticle sintering enables Ag-based inks to form reliable conductive contacts on polymer films, delivering classification accuracies above 90% for gesture recognition while preserving substrate integrity and enabling rapid, low-cost fabrication [257]. Complementary approaches employ photonic, room-temperature sintering to process Cu and Ag nanoparticle inks into low-resistivity nanomembrane electrodes and wireless circuits. Intense pulsed light promotes interparticle fusion while minimizing oxidation and thermal stress, enabling conformal, biocompatible EMG electrodes that match clinical-grade signal quality in multimodal wearable systems (Figure 6d,e) [254]. Beyond metals, chemically processed graphene inks form high-aspect-ratio, gel-free conductive networks without high-temperature annealing, providing stable, low-impedance skin interfaces for high-fidelity EMG recording and real-time human–machine control [255]. The performance of the printed electronics is demonstrated by using real-time control of external systems via electromyograms.

(3) Electrooculography (EOG)

Electrooculography (EOG) measures eye movements by detecting the voltage difference between the cornea and retina, providing a bioelectrical pathway for inferring user intent and enabling human–machine interaction. Low-temperature, additive fabrication was utilized to produce soft conductive electrodes that preserve mechanical compliance and skin compatibility. In one example, aerosol jet-printed Ag nanoparticle electrodes were patterned into an ultrathin, open-mesh architecture and transferred onto elastomeric substrates, forming low-impedance, stretchable dry electrodes without the need for gels or high-temperature post-processing [253]. These dry Ag nanoparticle electrodes are integrated with flexible wireless electronics to achieve real-time classification of eye vergence without gels or bulky hardware. The device is applied for virtual-reality–based ocular therapy, achieving high accuracy in monitoring convergence and divergence eye movements for conditions such as strabismus and convergence insufficiency.

(4) Electroencephalogram (EEG)

Electroencephalography (EEG) measures the collective electrical activity of neuronal populations by detecting faint voltage fluctuations at the scalp, enabling non-invasive monitoring of brain states for clinical diagnostics and human–machine interface applications. Low-temperature, soft-material processing strategies allow the integration of compliant, low-impedance electrodes into personalized and mechanically robust form factors. A recent article demonstrated EEG acquisition using 3D-printed smart electronic eyeglasses that integrate soft CNT/PDMS composite dry electrodes fabricated by elastomeric composite processing and embedded into a customizable PLA frame, enabling reliable, gel-free EEG recording and brain–computer interaction (SSVEP-based control) in a wearable, wireless format [259]. In another study, a fully portable brain–machine interface employs flexible scalp electronics with soft, skin-conformal dry electrodes and stretchable interconnects fabricated by thin-film microfabrication and low-temperature transfer printing of Ag nanoparticle networks onto elastomeric substrates. This strategy ensures low skin–electrode impedance and mechanical compliance without conductive gels, supporting stable multichannel EEG recording and real-time deep learning-based decoding of brain activity during motion [258]. The combination of these materials and processing strategies with embedded wireless electronics provided stable multichannel EEG recordings during motion, demonstrating a scalable route toward wearable EEG platforms for practical brain–machine interface applications [258].

(5) Photoplethysmography (PPG)

Recent advances highlight the critical role of low-temperature sintering inks in enabling conformal, skin-interfaced PPG devices. A PPG sensor is a non-invasive optical device that uses light (often green, red, or infrared LEDs) shining onto the skin to measure changes in blood volume, detecting blood flow fluctuations with a photodetector, commonly used in smartwatches and fitness trackers for heart rate, blood oxygen, and other vital sign monitoring by detecting light absorption/reflection changes with each heartbeat [250]. For example, an ultrathin epidermal PPG sensor was fabricated via direct ink writing of a stiffness tunable Ga–Cu composite ink, where the printed circuit remains mechanically robust during fabrication but undergoes softening at body temperature, allowing intimate and stable contact with the skin. This body temperature-driven transition, enabled by the low-temperature processability of the ink, eliminates the need for high-temperature sintering that would be incompatible with soft substrates and bio-integrated systems. As a result, reliable optical pulse sensing is achieved through integrated LED and photodiode circuitry on a stretchable SEBS substrate, demonstrating both mechanical adaptability and stable signal acquisition.

Moreover, these studies illustrate how low-temperature sintering and curing strategies are essential for integrating conductive materials with soft, temperature-sensitive substrates and biological interfaces. By preserving substrate integrity and maintaining bi-functional compatibility, such approaches enable high-performance printed and hybrid conductors to transition from proof-of-concept demonstrations to application-ready biosensor platforms spanning biochemical, biophysical, and electrophysiological domains. Across sweat, interstitial fluid, blood serum, microfluidic, transdermal, and cellular interfaces, key performance metrics, including sensitivity and bioenvironmental compatibility, now meet or exceed the requirements for wearable, implantable, and point-of-care sensing. Framed in this application-driven context, conductor integration strategies are best assessed by the reliability and longevity of the sensing functions they enable, rather than by the specific materials or processing routes employed, providing a flexible and robust foundation for next-generation biosensor technologies.

### 4.4. Design Guidelines for Biosensor Fabrication

Based on the examples discussed above, several general design principles can be identified for the fabrication of printed biosensors using low-temperature sintering approaches. First, ink formulations should be selected to balance electrical conductivity and processing temperature. Nanoparticle-based inks typically provide high conductivity but require effective ligand removal during sintering, whereas metal–organic precursor inks can enable lower processing temperatures due to in situ metal formation.

Second, the choice of sintering method should be guided by the thermal sensitivity of both the substrate and the biological functional layers. Photonic or laser sintering techniques are advantageous when rapid localized heating is required to avoid damaging flexible substrates or previously deposited bio-functional materials. Chemical sintering approaches may be particularly useful for enzyme-based biosensors where maintaining biological activity is critical.

Third, the desired electrode microstructure should be considered during process optimization. Dense conductive films are generally preferred for electrophysiological sensors requiring low noise and stable electrical contact, whereas partially porous electrodes may enhance sensitivity in electrochemical biosensors by increasing active surface area.

Finally, integration of bio-functional layers should be carefully designed to minimize exposure to high temperatures or reactive chemical environments during device fabrication. In most cases, conductive electrodes are printed and sintered prior to deposition of biological recognition elements to preserve their activity. Adopting these design strategies can significantly improve the performance and reliability of printed biosensors fabricated using low-temperature sintering technologies.

### 4.5. Challenges

While low-temperature sintering inks enable the fabrication of conductive features on flexible and biocompatible substrates, several challenges remain for their reliable use in biosensing applications. Many ink formulations rely on ligands, stabilizers, solvents, and reactive precursors to achieve densification below 200 °C or even at room temperature. Residual species from these components can remain in printed films after sintering, potentially affecting electrical stability, corrosion resistance, and biological compatibility when devices operate in physiological environments. In addition, the reduced thermal budget associated with low-temperature sintering also influences the microstructure of printed metals. Compared with high-temperature processes, films formed at low temperatures may contain higher porosity, incomplete ligand removal, or weaker particle necking, which can lead to increased resistivity, mechanical degradation, or metal ion release during long-term exposure to biofluids. These issues are particularly important for wearable and implantable biosensors, where conductive layers must maintain stable electrical performance under mechanical deformation and continuous contact with biological media.

At the same time, the need for low-temperature processing is driven by the integration of thermally sensitive substrates and biological components such as enzymes, antibodies, hydrogels, and ion-selective membranes. Processing conditions must, therefore, balance sufficient metal densification with preservation of biological functionality. Strategies such as optimized precursor chemistry, chemical sintering, encapsulation layers, and surface passivation are increasingly used to improve conductivity and stability while maintaining compatibility with soft substrates and bio-functional materials.

For practical deployment, these materials must also meet established biocompatibility and reliability standards for biomedical devices. Future research should, therefore, emphasize ink chemistries that minimize residual species, improve film stability under physiological conditions, and maintain high conductivity within the strict thermal limits required for bio-integrated electronics.

## 5. Outlook and Future Research Direction

Future research opportunities in low-temperature sintering inks are closely aligned with the rapid expansion of wearable and implantable biosensor platforms. Emerging applications such as continuous metabolic monitoring, neural interfaces, and epidermal health diagnostics require highly reliable electronic components that can operate on soft, deformable substrates for extended periods. Advances in room-temperature self-sintering inks, liquid metal conductors, and biocompatible conductive polymers may enable fully printed bioelectronic systems that integrate sensing, signal processing, and wireless communication within a single platform. Additionally, scalable manufacturing approaches such as roll-to-roll printing and photonic curing will be essential for translating laboratory-scale biosensors into commercial healthcare technologies. This review highlights low-temperature sintering inks as foundational materials enabling printed electronics for biosensing, wearable, and bio-integrated systems. The unifying principle is that ink chemistry, formulation, and sintering strategy must be co-designed to achieve high electrical performance under stringent thermal, mechanical, and biological constraints. This principle applies across diverse material systems, including metal nanoparticle inks, particle-free metal–organic decomposition (MOD) inks, metal oxides, chalcogenides, liquid metals, and hybrid material systems. Advances in ligand chemistry, precursor design, ink formulation, and sintering pathways have collectively pushed processing temperatures well below 200 °C, and in some cases to room temperature. Below, we list potential research directions to address the major challenges.

### 5.1. Performance–Processing Tradeoffs

Despite this progress, several critical challenges remain that limit the translation of laboratory demonstrations to scalable manufacturing and long-term deployment. Many of these issues remain insufficiently addressed in the current literature and represent important directions for future research. Key challenges include multidimensional tradeoffs among electrical performance and substrate thermal integrity, cost efficiency and device sensitivity, and manufacturing throughput and energy consumption. Achieving high conductivity remains challenging when constrained by substrate damage, as the thermal energy required for metal densification must remain below the glass transition temperatures of flexible materials such as PET or paper. While many room-temperature or chemically sintered systems achieve acceptable conductivity, they often suffer from long-term instability, environmental sensitivity, or mechanical degradation under repeated deformation.

From a processing cost versus performance perspective, although Ag inks provide superior conductivity, their high cost motivates increasing exploration of alternatives such as Cu or Ni. These materials may offer slightly lower performance but significantly reduce material costs. Furthermore, since conventional thermal sintering remains the most cost-effective approach compared with advanced alternatives, current research increasingly focuses on particle-free formulations that can be converted at substantially lower temperatures. The tradeoff between energy consumption and throughput is also being addressed through rapid sintering techniques such as photonic or microwave processing, which enable high-volume manufacturing but require precise pulse control to prevent thermal stress at material interfaces. Addressing these multidimensional constraints will be essential for the next generation of resilient printed biosensing systems.

### 5.2. Materials Sustainability and Cost

Material sustainability and cost considerations also remain relatively underexplored. Ag continues to dominate low-temperature conductive inks due to its straightforward chemistry and resistance to oxidation; however, its cost and supply constraints motivate increased attention toward Cu-based systems, hybrid materials, and biobased stabilizers. Although significant progress has been achieved in Cu MOD inks and oxidation-resistant ligand systems, the development of fully air-sinterable, room-temperature Cu inks with a long shelf life and high reliability remains an open challenge. The incorporation of biobased polymers and green solvent systems represents an important future direction toward more sustainable printed electronics.

### 5.3. Integration and Multilayer Device Complexity

Integration complexity represents another major barrier for multifunctional and multilayer biosensing systems. Real-world wearable and implantable devices require hierarchical architecture combining conductors, dielectrics, semiconductors, encapsulants, and bio-functional layers. Consequently, low-temperature sintering processes must remain compatible not only with substrates but also with adjacent functional layers and sequential processing steps. Future research may, therefore, prioritize orthogonal sintering strategies, selective energy delivery, and process windows that enable multilayer fabrication without cross-layer degradation or delamination.

### 5.4. Industrial Translation and Manufacturing Scalability

As printed biosensors move toward commercialization, several key industrial factors must also be addressed. Roll-to-roll (R2R) compatibility is currently a major driver for nanoparticle-based inks, as their rapid sintering kinetics under photonic or infrared sources enable high-speed, large-area manufacturing. In contrast, state-of-the-art particle-free (MOD) inks are increasingly favored for digital printing techniques such as inkjet or aerosol jet printing due to their favorable rheology and reduced risk of nozzle clogging, making them particularly suitable for the high-resolution and complex geometries required in biosensing applications. The commercialization of several Ag and Cu MOD ink systems already demonstrates their potential, particularly due to their improved shelf stability compared with traditional nanoparticle formulations.

### 5.5. Emerging Opportunities in Materials and Data-Driven Design

Low-temperature sintering inks will likely benefit from the convergence of multiple technologies rather than from singular material advances. Hybrid ink systems that combine metallic conductivity with polymeric compliance, liquid-metal fluidity, or nanocarbon reinforcement may help overcome limitations associated with single-component inks. Looking forward, the evolution of low-temperature sintering inks will increasingly be driven by the integration of machine learning (ML) and artificial intelligence (AI). Data-driven approaches are beginning to replace traditional trial-and-error methods in ink formulation, enabling rapid optimization of complex precursor–ligand interactions to achieve targeted rheology and performance with fewer experimental cycles. At the same time, the growing demand for bio-integrated devices is driving the development of ultra-low-energy sintering techniques. Methods such as flash sintering minimize the thermal budget, thereby protecting delicate protein-based or hydrogel-based substrates. Achieving high-purity metallic films at or near room temperature is essential for maintaining electrochemical stability in sensors that operate in prolonged contact with biological fluids or tissues. Similarly, combining steady-state thermal sintering with localized photonic, laser, chemical, or electrical activation provides a pathway to balance uniformity, throughput, and substrate safety. Emerging concepts such as self-sintering inks, cold-sintering-assisted printed layers, and stimulus-responsive or phase-change inks further expand the design space for bio-integrated electronics.

In addition, application-driven performance metrics should guide future ink and process development. For biosensing applications, performance should be evaluated not only by conductivity or pattern resolution, but also by signal fidelity, impedance stability, biocompatibility, mechanical durability, and overall system-level reliability. As printed electronics move closer to clinical, consumer, and industrial deployment, standardized testing protocols, long-term aging studies, and failure-mode analyses will become as important as materials innovation itself.

In summary, low-temperature sintering inks are evolving from more fundamental research into enabling technologies for next-generation printed bioelectronics. Continued progress will rely on interdisciplinary advances spanning chemistry, materials science, device engineering, and manufacturing. By aligning ink design with realistic application requirements and scalable processing strategies, low-temperature sintering inks will play a central role in the future development of biosensors, wearable devices, implantable systems, and soft electronics.

## Figures and Tables

**Table 1:** Composition, sintering temperature, resistivity, and key features for metal inks.

Material	Sintering Temperature (°C)/References	Resistivity	Key Features
Ag (nanoparticle based)	25 [82,83,84,85,86]	~3.84–15.7 µΩ·cm (~2–10× bulk Ag)	Chemical sintering, no external heating required, compatible with temperature-sensitive substrates
	150–350 [9,78]	~2.8–6 µΩ·cm (~2–4× bulk Ag)	HEC capping agent, biobased polymer, high solids loading (40 wt%), good bending endurance
	>300–350 [5,87,88,89,90]	~5–14.2 µΩ·cm (~3–10× bulk Ag)	PVP capping agent, commercially common, robust, higher thermal budget
Ag (particle free)	25–200 [61,62,91,92]	~1.5–4.8 µΩ·cm (~1–2× bulk Ag)	Reactive Ag metal–organic decomposition (MOD) (oxalate/diamine), particle free, reduced printing issues, no polymer dispersant required
Cu (particle free)	130–180 [39,93,94,95,96,97,98,99,100]	~3–5 µΩ·cm (~2–3× bulk Cu)	Cu MOD (formate + amine/alkanolamine), lower T than Cu nanoparticles, ligand design controls oxidation
	≤200 [98,100]	~3–10 µΩ·cm (≈2–6× bulk Cu)	Air-sinterable Cu precursor (mixed ligands), dual-/multi-ligand strategy, improved air stability
Gold (particle free)	150–280 (thermal); Room temperature (photo) [101,102,103,104]	~5–10 µΩ·cm (~2–4× bulk Au)	Au MOD or HAuCl_4_-based ink, excellent chemical stability and biocompatibility
Nickel (particle free)	>200 (or ~180 with dual-ligand) [105,106]	~50–150 µΩ·cm (~7–20× bulk Ni)	Ni MOD (formate-amine complex), self-reducible in inert atmosphere; dual-ligand lowers T

**Table 2:** Low-temperature sintering methods, their advantages, and their limitations.

Methods	Advantages	Limitations	Applications
Thermal Curing: 60–150 °C; 30 min typical	Uniform performance; conductivities 30–40% of bulk Ag; resistivities as low as 13.38 µΩ·cm	Residual porosity; skin formation risk; requires careful temperature control	Flexible electronics; conductive circuits [178,179,181,182,183]
Photonic Sintering: Xenon flash lamps; >500 °C (mili second pulses)	High-speed processing: substrate remains cool; enables roll-to-roll manufacturing	Dependent on ink optical properties; limited for wide-bandgap materials	High-throughput manufacturing; roll-to-roll processing [184,185,186,187,188]
Laser Sintering: Selective beam scanning; variable power/speed	Spatial selectivity; micro-scale patterning; substrate-friendly	Heat-affected zone in multilayers; wavelength-dependent efficiency	Micro-patterns; selective metallization [189,190,191,192,193]
Plasma Sintering: Pulsed electric field; seconds timescale	Ionic stripping removes oxides; near-theoretical densities; rapid mass transport	Requires specialized equipment; limited to powder compacts	Ceramics densification [194,195]
Microwave Flash Sintering: seconds timescale	Exponential energy absorption; instantaneous densification	Requires threshold temperature; thermal runaway control needed	Ceramics densification [194,196]
Chemical Sintering: RT–200 °C; reducing agents, acids, chlorides	Room-temperature capable; 20% bulk Ag conductivity; compatible with plastics	Cu oxidation sensitivity; requires controlled atmosphere for some systems	Temperature-sensitive substrates; biosensors [53,82,83,84,85,86,96,197,198,199,200,201,202]
Cold Sintering Process: <300 °C; transient liquid phase; low pressure	Dissolution–precipitation mechanism; near-theoretical densities	Requires pressure application; limited material compatibility	Ceramics; composite co-processing [180]
MOD Ink: RT; self-decomposing Ag complexes	No thermal processing; highly conductive; thermally sensitive substrate compatible	Specialized ink formulation required; limited to reactive metal complexes	Wearable biosensors; electronic skin [92]
Liquid Metal: RT; no post-processing required	Fluidic bridging; 3D surface adaptable; no sintering needed	Limited material selection; oxide layer management needed	Stretchable electronics; variable stiffness devices [173,174,175,203]
Solution Sintering: Reactive binders (e.g., VegPU)	350% stretchability; ~12,833 S/cm conductivity	Binder chemistry optimization; interfacial reaction control required	Soft electronics [178,181]
Electrical Sintering: Applied electric field	Rapid localized heating; energy-efficient	Requires conductive pathways; potential for non-uniform heating	Nanoparticle structures [38]

**Table 3:** Comparison of low-temperature sintering methods for printed biosensor fabrication.

Sintering Method [References]	Temperature (°C)/Energy Input	Processing Time	Microstructural Characteristics	Advantages	Limitations
Thermal [90,179,193,205]	80–200 °C (convection, hotplate, IR)	Min-Hrs.	Gradual particle neck growth; moderate porosity; relatively uniform films	Simple processing scalable for roll-to-roll manufacturing; good film uniformity	Long exposure to heat may damage polymer substrates or degrade biomolecules
Photonic [184,185,187,188]	Xenon flash lamp pulses (>500 °C locally, ms duration)	Milli-seconds (ms)	Rapid nanoparticle fusion; dense surface layer; minimal substrate heating	Compatible with flexible substrates; extremely fast processing; high conductivity	Requires strong optical absorption of inks; possible thermal stress and cracking
Laser [88,93,125,189,190]	Localized laser heating	Seconds	Highly localized densification; precise microstructures	Spatial selectivity enables multilayer device fabrication without damaging adjacent bio-layers	Equipment complexity; limited throughput for large-area devices
Chemical [30,82,84,175]	RT–150 °C	Min	Particle coalescence via ligand removal or surface reduction; sometimes porous structures	Excellent compatibility with bio-functional layers; suitable for enzyme-based biosensors	Possible chemical residues; control of reaction uniformity required
Plasma [54,121,122,183,194,206]	Low bulk temperature; plasma-activated surfaces	Seconds	Surface oxide removal; enhanced particle diffusion; dense metallic networks	Rapid processing; improved conductivity for oxide-contaminated nanoparticles	Plasma exposure may degrade organic functional layers or polymer substrates
Microwave sintering [207,208]	Rapid dielectric heating	Seconds–minutes	Internal volumetric heating; rapid densification	Energy efficient; fast heating rates	Non-uniform heating depending on material dielectric properties

**Table 6:** Various printed wearable devices for detecting physiological signals using low-temperature sintering inks.

Electrode Material	Fabrication Method and Sintering Temp.	Substrate and Sensing Mechanism	Key Features	Application and References
Ag flakes	Screen printing; 80 °C	Textile; ionic conduction at electrode–tissue interface	Rubbery stretchability-gradient substrate stretched to 110%, and a wearable electromyogram sensor printed onto a textile garment	EMG device [112]
Ag/AgCl	Screen printing; 80 °C	Textile; electrochemical bio-potential detection	Wearable devices track the wearer’s physicochemical, electrophysiological status.	ECG and sweat-lactate levels [252]
Ag	Aerosol jet printing; 130–200 °C 200 °C best	Elastomer; eye tracking in virtual reality	Ultrathin, aerosol jet–printed Ag nanoparticle EOG electrode system that is soft, stretchable, and wireless, enabling high-accuracy real-time eye vergence detection for VR-based ocular therapy	EOG [253]
Cu NP and Ag NP	Aerosol jet printing; IPL sintering	Polyimide; electrochemical bio-potential detection	Detect multiple physiological signal	EMG, ECG, EOG, motions [254]
Functionalized conductive graphene ink	Aerosol jet printing; 100 °C (active material) 250 °C (curing the substrate)	Elastomer; ionic conduction at electrode–tissue interface	key technological advancements are the use of a functionalized conductive graphene with enhanced biocompatibility, anti-oxidation, and solderability, which allows a wireless flexible circuit.	EMG [255]
Commercial Ag flakes	Screen printing; 60–80 °C	Elastomer; amperometric detection using lactate oxidase	A fully in-ear integrated sensor for monitoring brain-state and dynamic lactate-concentration changes for the detection or monitoring of neurodegenerative diseases.	EEG, EOG, and electrodermal activity, lactate in sweat [256]
Ag NP	Inkjet printing; RT Chemical sintering	Elastomer; ionic conduction at electrode–tissue interface	Multi-channel sEMG signals ensured consistent values across repetitions in every participant	EMG [257]
Cu/Ga composite ink (bulk Ga and Cu powder)	Screen printing; 50 °C	PI; PPG signal	ultrathin epi-dermal PPG sensor and a wireless optoelectronic device capable of converting between flexible and rigid configurations for biomedical applications.	PPG [250]
Ag NP	Aerosol jet printing; 50 °C	PDMS; ionic–electronic coupling at electrode–skin interface	fully portable and wireless brain–machine interface in which flexible scalp electronics	EEG [258]
CNT/PDMS	Screen printing; 60–80 °C	Elastomer; amperometric detection using lactate oxidase	3D-printed smart eyeglasses	EEG, EOG [259]
Ag NW	Screen printing; 80 °C	Elastomer; electrochemical bio-potential detection	Fully screen-printed wet ECG electrodes for both monitoring and diagnostic purpose.	ECG [244]
Ag NWs/GO	Screen printing; 130 °C	Elastomer; electrochemical bio-potential detection	Fully screen-printed transparent wet ECG electrodes	ECG [113]

## Data Availability

No new data were created or analyzed in this study.
